# Complete chloroplast genome sequence of MD-2 pineapple and its comparative analysis among nine other plants from the subclass Commelinidae

**DOI:** 10.1186/s12870-015-0587-1

**Published:** 2015-08-12

**Authors:** R. M. Redwan, A. Saidin, S. V. Kumar

**Affiliations:** Biotechnology Research Institute, Universiti Malaysia Sabah, Jalan UMS, 88400 Kota Kinabalu, Sabah Malaysia; Novocraft Technology Sdn. Bhd., 3 Two Square, Seksyen 19, Petaling Jaya, Selangor Malaysia

## Abstract

**Background:**

Pineapple (*Ananas comosus* var. *comosus*) is known as the king of fruits for its crown and is the third most important tropical fruit after banana and citrus. The plant, which is indigenous to South America, is the most important species in the Bromeliaceae family and is largely traded for fresh fruit consumption. Here, we report the complete chloroplast sequence of the MD-2 pineapple that was sequenced using the PacBio sequencing technology.

**Results:**

In this study, the high error rate of PacBio long sequence reads of *A. comosus*’s total genomic DNA were improved by leveraging on the high accuracy but short Illumina reads for error-correction via the latest error correction module from Novocraft. Error corrected long PacBio reads were assembled by using a single tool to produce a contig representing the pineapple chloroplast genome. The genome of 159,636 bp in length is featured with the conserved quadripartite structure of chloroplast containing a large single copy region (LSC) with a size of 87,482 bp, a small single copy region (SSC) with a size of 18,622 bp and two inverted repeat regions (IRA and IRB) each with the size of 26,766 bp. Overall, the genome contained 117 unique coding regions and 30 were repeated in the IR region with its genes contents, structure and arrangement similar to its sister taxon, *Typha latifolia*. A total of 35 repeats structure were detected in both the coding and non-coding regions with a majority being tandem repeats. In addition, 205 SSRs were detected in the genome with six protein-coding genes contained more than two SSRs. Comparative chloroplast genomes from the subclass Commelinidae revealed a conservative protein coding gene albeit located in a highly divergence region. Analysis of selection pressure on protein-coding genes using Ka/Ks ratio showed significant positive selection exerted on the *rps7* gene of the pineapple chloroplast with P less than 0.05. Phylogenetic analysis confirmed the recent taxonomical relation among the member of commelinids which support the monophyly relationship between Arecales and Dasypogonaceae and between Zingiberales to the Poales, which includes the *A. comosus*.

**Conclusions:**

The complete sequence of the chloroplast of pineapple provides insights to the divergence of genic chloroplast sequences from the members of the subclass Commelinidae. The complete pineapple chloroplast will serve as a reference for in-depth taxonomical studies in the Bromeliaceae family when more species under the family are sequenced in the future. The genetic sequence information will also make feasible other molecular applications of the pineapple chloroplast for plant genetic improvement.

**Electronic supplementary material:**

The online version of this article (doi:10.1186/s12870-015-0587-1) contains supplementary material, which is available to authorized users.

## Background

Pineapple is an old fruit, in the sense that it has a long history of human consumption. The flesh was described as “the most exquisite fruit in existence” by Pigafetta in 1519 (reviewed in [1]) and to the present it is still highly regarded for its sweet and tart flavour. Mostly consumed fresh, the fruit is not only appreciated as a gastronomic pleasure, but is also used as a digestive aid taken between meals, as well as a meat tenderizer due to the presence of a strong protease enzyme known as bromelain [2]. Traditionally, different varieties of the *Ananas* species have been grown to make alcoholic beverages, poison and the high fibre leaves of the plant was used as fabric for clothing [3]. *Ananas comosus* is from the Bromeliaceae family in the order of Poales and the subclass of Commelinidae. The family consists of 56 genera and over 2885 species [4]. However, as of 11 February 2015, there was no entry of complete plastid sequence in NCBI Organelle Genome Resources database for any species from the family. Despite of that, multiple studies have been carried out to study the relationship among the members of the family by using multiple short sequences derived from the chloroplast [5–8]. The chloroplast sequence carries important information for plant molecular systematics to study the taxonomic classification of plants. With the use of molecular data in inferring phylogenic relationships among plants, many reclassifications have occurred and new orders are being recognized [9]. This includes the recognition of the new commelinids clade which contains the family Bromeliaceae.

Initially, the discovery of the chloroplast DNA sequence began with the physical mapping of the *Zea mays* chloroplast which was produced by digestion with multiple restriction enzymes [10]. Later, the first complete nucleotide sequence of *Nicotiana tabacum* was unravelled by a tedious clone by clone sequencing of the plasmid and cosmid libraries [11]. This was followed by chloroplast sequences for several plant species [12–14]. Spearheaded by the sequencing of pooled chloroplast genomes of the pine family [15], more chloroplast genomes have been sequenced using next generation sequencing (NGS) techniques such as the massive parallel sequencing and pyrosequencing. However, the *de novo* assembly of these millions of NGS reads proved to be a cumbersome process. The technique usually calls for a reference-guided assembly followed by the *de-novo* assembly of the short reads as it could not be overlapped to each other independently [16–18]. The pyrosequencing technology reads however were successfully utilized to form a single contig by iterative mapping of the read ends but the draft is prone to homopolymer error which required additional low-throughput Sanger sequencing for assembly correction [19]. Recently, several chloroplast genomes were published using long read sequencing technology from PacBio [20–23]. In these studies, the plastid genome sequences were assembled to a final single contig of finished genome. These achievements highlighted the usefulness of long read sequences for *de novo* assembly and in general many genomes have been successfully assembled using PacBio [24–26].

Chloroplast genes have often been used to infer plant phylogeny at different taxonomic orders. The most notable is the recognition of the commelinids clade which is supported by both molecular and morphological data as recognized by the Angiosperm Phylogeny Group [9]. The clade was grouped into five orders based on the uniformity of the molecular data [27] and the co-presence of UV-fluorescent ferulic acid bound cell walls [28]. In a recent study, Barrett et al. [29] managed to used multiple chloroplast genes to resolve the orders among the commelinids clade and their results showed a robust support of relationship between Arecales and Dasypogonalesas as sisters to Zingiberales, Commelinales and Poales. This study aims to characterize the pineapple chloroplast genome and to compare the pineapple chloroplast to other members of the commelinids clade by using PacBio error corrected reads to perform *de novo* assembly of the chloroplast genome. We also hope to share with the readers the benefits and challenges faced when dealing with the large inverted repeat region of the chloroplast.

## Material and methods

### Sample materials

The world renowned MD-2 pineapple hybrid was selected for sequencing due to its high quality fruit and worldwide demand. The pineapples were obtained courtesy of the Malaysian Pineapple Industry Board. The MD-2 was initially developed through the Pineapple Research Institute (PRI) breeding program in the 1960s. The line was later released to the Maui Pineapple Company and Del Monte for further evaluations in the 1980s. MD-2 was derived from a cross of two PRI hybrids resulting in a complex mixture of several pineapple varieties but with a high proportion of the Smooth Cayenne, which was the dominant pineapple variety at that time.

### Illumina library construction and sequencing

Total genomic DNA was extracted from fresh pineapple leaves according to the protocol of Carlier et al. [30] with slight modifications. Briefly, pineapple leaves were ground with liquid nitrogen into powdery form. The powder was then mixed into preheated 5 mL extraction buffer containing 200 mM Tris–HCl pH 8; 25 mM EDTA; 250 mM NaCl; 1 % SDS and 2 % PVP. The mixture was then incubated at 65 °C for 30 mins with 10 mins mixing intervals. Next, 10 μL of RNAse (100 mg/mL) was added and the incubation was continued for another hour at 37 °C. Subsequently, 5 μL of proteinase K (20 mg/mL) was added and incubated for another 30 mins at 55 °C. The mixture was purified to remove protein contamination by successive extraction using phenol:chloroform:isoamyl alcohol organic solvent and was followed by the addition of 1/10 volume of 2 M potassium acetate and equal volume of isopropanol to the aqueous solution to precipitate the DNA. Finally the crude DNA sample was washed with two rounds of freshly prepared 70 % alcohol and diluted in 100 μL TE buffer.

The integrity of the DNA sample was inspected using 1 % agarose gel electrophoresis while its purity was measured at A260/A280 and A260/A230 ratio, respectively, by using a spectrophotometer (Nanodrop 2000, Thermo Scientific). The concentration of the DNA was measured using a fluorometer (Qubit 2.0, Life Technologies) and 10 μg of total DNA was sequenced using the Illumina HiSeq on one lane with 100 bp paired end format. The sequencing service was provided by Macrogen, Korea. For library preparation, TruSeq PCR Free (Illumina, USA) library preparation was used according to the manufacturer’s protocol. The short reads received were then quality-trimmed and length-filtered using fqtrim software (https://ccb.jhu.edu/software/fqtrim/index.shtml) to a minimum quality of Q20 and length of 50 bp and above, as well as in conjunction to the sequencing adaptor removal. High quality reads with a total size of 38 GB was produced with only 8.87 % of total data discarded.

### PacBio library construction and sequencing

When performing real-time single molecule sequencing using the PacBio RSII, it is crucial that the DNA purity is high in order to guarantee good sequencing performance. Conventionally, the combination of phenol, chloroform and isoamyl alcohol is used in order to denature protein contamination during DNA extraction as in the previously described method of Carlier et al. [30]. However, the use of the strong solvent could potentially cause organic substance carryover that reduces the purity of the extracted DNA. Thus, alternative methods such as the salting out protocol as described by Dellapota et al. [31] was adopted. Similarly, the fresh leaves were crushed in liquid nitrogen. Next, 5 ml of extraction buffer (1 % β-mercaptoethanol, 100 mM Tris pH 8, 50 mM EDTA pH 8 and 500 mM sodium chloride), and 330 mL of 20 % SDS was added to the powdered leaves. The mixture was then incubated at 60 °C with mixing at 950 rpm for 10 mins on a Thermomixer (Eppendorf, USA). Next, 5 μl of RNase A (100 mg/mL) was added and incubation continued at 37 °C on Thermomixer with mixing at 500 rpm for an hour. Then, 1.6 mL of 5 M potassium acetate was added and incubation was continued for half an hour on ice. The mixture was then centrifuged at 13,000 rpm for 20 mins and only the supernatant was collected into a fresh tube containing 3.30 mL of isopropanol. The mixture was then inverted and incubated overnight at −20 °C. The DNA sample was then pelleted by centrifugation at 10,000 rpm for 30 mins and the pellet was then re-suspended in 500 μl TE buffer. After the pellet had fully dissolved, 75 μl of 3 M sodium acetate and 500 μl of isopropanol were added and the tube was inverted multiple times to mix. The tube was then incubated in −80 °C for 20 mins and was centrifuged at 10,000 rpm for 30 mins. The crude DNA was then washed with 70 % alcohol twice before it was re-dissolved in 50 μl TE buffer.

The integrity of the DNA was inspected using 1.0 % agarose gel electrophoresis and the purity index was measured using Nanodrop 2000 Spectrophotometer for A260/A280 and A260/A230 ratios. Both indexes were measured at 1.9 and 1.8, respectively. SMRTbell DNA Template Prep Kit v.1 (Pacific Biosciences, USA) in conjunction with the P4-C2 and P5-C3 sequencing chemistry were used to construct libraries according to PacBio Sample Net-Shared Protocol, available at http://pacificbiosciences.com/. The libraries were sequenced using the PacBio RSII and a total of 6655 MB was generated. The average read length for PacBio sequencing reads was 5306 bp with the longest read at 37,591 bp long. The PacBio reads were then processed using NovoCleaverLR from Novocraft which functions to align and subsequently removes any presence of SMRTBell adaptor sequences in the filtered subreads of the PacBio. This yielded 873,181 high quality reads of 4.6 GB in total size. The cleaned reads were then corrected using both the 350 bp and 750 bp Illumina high quality short reads through mapping by using the Novoalign program. The NovoCorrector was then applied to the alignment file produced to perform variant call and to produce error-corrected PacBio reads.

### Genome assembly

Four hundred and seventy available chloroplast genomes (Additional file 1) were downloaded from the NCBI database and these sequences were used to find read sequences which are similar to the pineapple chloroplast by using GMAP aligner at default setting [32]. Using the chloroplasts as database, 818,142 long error-corrected PacBio reads were queried through the GMAP aligner. The psl alignment file from GMAP output was processed using in-house script in order to generate percent identity score and alignment length. Altogether, a number of 58,126 unique reads had hits to the chloroplast database. From this, 41,188 reads were selected based on the alignment length of more than 100 bp and a minimal read length of 1000 bp was used in the *de novo* assembly of the chloroplast genome of pineapple. MIRA v4.0.2 [33] was used in order to build contigs from the 41,188 long chloroplast error corrected PacBio reads which employed an overlap graph algorithm and automated genome finishing. Initial assembly of the 41,188 error-corrected PacBio reads (with a total size of 182 Mb) using MIRA v4.0.2 yielded 246 contigs with N50 of 14,757 bp. The two largest contigs with a size of 95,865 and 64,927 bp in length had similar GC content (36 %) and both had approximately the same reads coverage (108–118×) compared to other contigs. From the contigs assembled, two largest contigs with similar GC percentage were used as baits to once again capture reads belonging to the chloroplast sequence from the full error corrected PacBio reads using the GMAP aligners, set at default. For this round, only hits with percent-identity of more than 70 % and length of alignment in match above 1000 bp were selected and this gave 19,260 reads. These reads were then once again processed by MIRA using the same parameters for *de novo* assembly, producing a single contig size of 164,813 bp. Upon alignment to the *Typha latifolia* complete chloroplast genome (GenBank: NC_013823.1), the contig was then manually rearranged to form the conserved quadripartite structure. In detail, the contig was truncated into two fragments and were merged using Megamerger (http://emboss.bioinformatics.nl/cgi-bin/emboss/megamerger) to find overlapping regions and to further merge it into a sequence. This merged fragment was later loaded into SMRTPortal as reference and the raw reads from the PacBio were mapped back to the assembled chloroplast for validation using BRIDGEMAPPER_RS and base correction by Quiver.

For evaluation purposes, all of the reads from the uncorrected filtered PacBio reads (873,181 reads) in total of 4.6 Gbp, and the corrected PacBio reads (821,079 reads) in total of 3.14 Gbp were mapped back to the final chloroplast genome using blasr long alignment (−bestn 5 –minPctIdentity 90 –placeRepeatsRandomly) [34].

### Genome annotation

The assembled chloroplast sequence was annotated using Dual Organellar Genome Annotator (DOGMA) [35] with manual start and stop codon validation by using the Sequin tool from NCBI (http://www.ncbi.nlm.nih.gov/Sequin). Next, tRNAScan-SE [36] was used to annotate tRNA using organellar search mode with Cove cut-off score of 15. Forward and inverted repeats were identified using REPuter [37] with minimal repeat size of 30 bp, hamming distance of 3 and with identity of no less than 90 %. Nucleotide frequency, and Relative Synonymous Codon Usage (RSCU) [38] were analysed using DAMBE [39] on the 83 protein-coding genes and only genes in IRA were used to represent the repeated genes. The circular genome was drawn using the OGDRAW program [40].

### Simple Sequence Repeats (SSRs) identification

Simple sequence repeats (SSRs) were identified using MISA (http://pgrc.ipk-gatersleben.de/misa/misa.html) with the following parameters; minimum SSR motif length of 8 bp and repeat length of mono-8, di-4, tri-4, tetra-3, penta-3 and hexa-3. MISA also identified compound SSR which are SSRs that are in adjacent, separated by certain length of sequences. In this analysis, the maximum size of interruption allowed between two different SSRs in a compound SSR was 100 bp.

### Genome comparison

One species was selected from every order under the subclass Commelinidae, except for the Poales, in which a single species was selected to represent each clade (BEP, PACMAD, Pueliodeae, Pharoideae, Anomochlooideae and Typhaceae). Under the order of Poales, *Olyra latifolia*, *Aristida purpurea*, *Puelia olyriformis*, *Pharus lappulaceus* and *Anomochloa marantoidea* were chosen to represent the graminids (Poaceae). Also included in the order of Poales was *T. latifolia*, to represent the Typhaceae family, which is known to be the sister clade of Bromeliaceae [29] and hence, is the closest to the pineapple chloroplast genome. Other chloroplast genomes included were *Ravenala madagascariensis* to represent the order Zingiberales, *Calamus caryotoides* for the order Arecales and *D. bromeliifolius* for the family Dasypogonaceae. All nine chloroplast genomes were compared to the pineapple chloroplast genome using NCBI BLASTX tool via BLAST Ring Image Generator (BRIG) at default settings [41]. A close inspection of specific sequences for pairwise comparison of the listed species to the pineapple chloroplast genome was aided by the Artemis Comparison Tool (ACT) [42] and the comparison file required for the tool was produced by Double ACT v2 (http://www.hpa-bioinfotools.org.uk/pise/double_act.html).

### Phylogenetic analysis

As of February 2015, there were 108 plastid genomes under the subclass of Commelinidae in the NCBI Organelle Genome Resources. From these, 100 taxa were chosen for further phylogenetic analysis; the other eight were excluded as they have more than ten genes either missing or unannotated. A total of 80 protein-coding gene sequences from all 100 taxa and pineapple were aligned; *orf48*, *orf56* and *ycf15* were excluded as they were not present in any other commelinids chloroplast genome. For each species, the 80 protein-coding gene sequences were translated to amino acids, were aligned using MAFFT aligner tool [43] and finally, the respective nucleotide sequences were aligned back based on the translated amino acids’ alignment using back-translator tool from TreeBeST (http://treesoft.sourceforge.net/treebest.shtml). Average pairwise sequence divergences for each gene were calculated using Kimura two-parameter (K2P) model using Mega 6 [44]. The Ka/Ks value for each gene was calculated using the KaKs_calculator at default setting (−c 11) [45]. Additionally, to test the significance of positive selection on the *rps7* gene, all the sequences available from the 100 taxa were aligned as above and analysed using Codon Based Z-test of positive selection from Mega 6 [44]. For phylogenetic analysis, 56 protein-coding gene sequences common to all 100 taxa were concatenated sequentially and were aligned as above. The aligned nucleotide was then tested in DAMBE for saturation of substitution and this resulted in Iss < Iss.c at *p* less than 0.05, which suggested that there was no saturated sites in these sequences. The sequences were then analysed using jModelTest 2.1.7 [46] to find the most optimal model with the lowest AICc value and the model chosen was the general time reversible (GTR) model with rate variation among sites and invariable sites. The phylogenetic tree was inferred by using maximum likelihood method with rapid bootstrapping of 1000 replicates and model of binary, GTRCAT which utilized the discrete approximation of the gamma distribution under RAxML version 8 tool [47]. The final nucleic acid alignment used is shown in Additional file 2. Both the alignment and maximum likelihood analysis were performed using the CIPRES Science Gateway v3.3 [48].

## Results and discussion

### Genome assembly

**Fig. 1:**
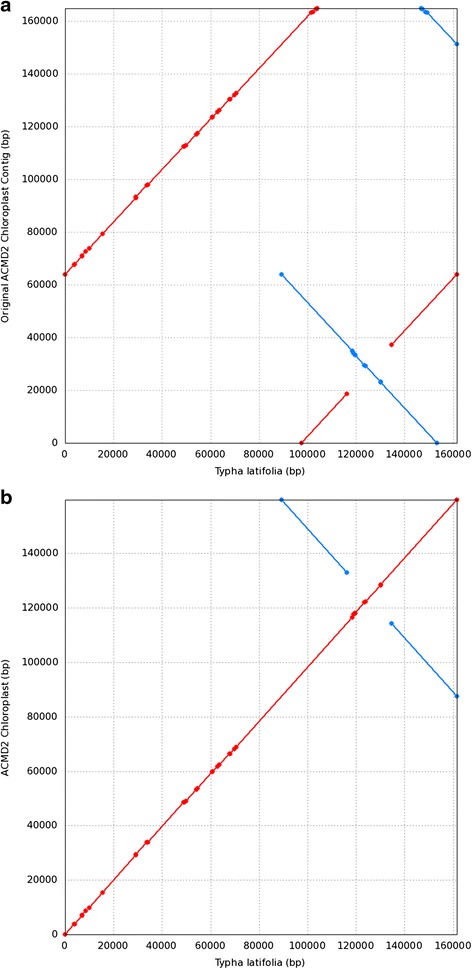
Syntenic dotplot generated by MUMmer [96] based on nucmer alignment between the contig produced by MIRA (**a**) before rearrangement and (**b**) after rearrangement to the *T. latifolia* chloroplast genome as the reference. The initial contig produced by MIRA had a different start as compared to conserved chloroplast structure such as the *T. latifolia* and it was in overlap to its ends which was the inverted repeat region. In addition, the SSC was in inverse as compared to the reference due to the uncertainty in placing the first copy of the repeated sequence flanking the SSC

Initial assembly of chloroplast long sequence reads via MIRA produced a single contig with a size of 164,814 bp. However, we recognized two problems with the contig when it was mapped to its closest relative, *T. latifolia* (Fig 1). Firstly, the starting and ending of the contig was found to be different from the reference genome (i.e. *T. latifolia* chloroplast genome). This issue, however, was not a major concern as it was easily resolved through manual rearrangement based on the reference. Secondly, the short single copy (SSC), which was flanked by large inverted repeats, was in the inverse orientation when compared to the reference sequence. Although the contig had a correct arrangement in a sense that the SSC was flanked by two identical sequences (i.e. inverted repeat copies), the orientation of the SSC could not be determined unambiguously. The inverted repeat sequences were identical to each other, with sizes of more than 26 kb each and separated by only a short sequence of 18 kb in length. Thus, to solve this, an assembler would require reads that extend through the repeats, start and stop only in the unique copy of the chloroplast (i.e. LSC/SSC). Any reads that starts in the SSC and ends in the repeat region would be ambiguously placed in either of the repeat copy and would cause ambiguity in the placement of repeats and thus the orientation of the SSC. For a read to contain the repeat as described, it should have a length of more than 30 kb, but the maximum length of the error corrected sequence reads used in the assembly was only 24,950 bp. Thus, to resolve the issue, the contig was broken up and reassembled by Megamerger as along with manual rearrangement to follow the conserved structure of the published chloroplast genomes of the land plants.

**Fig. 2:**
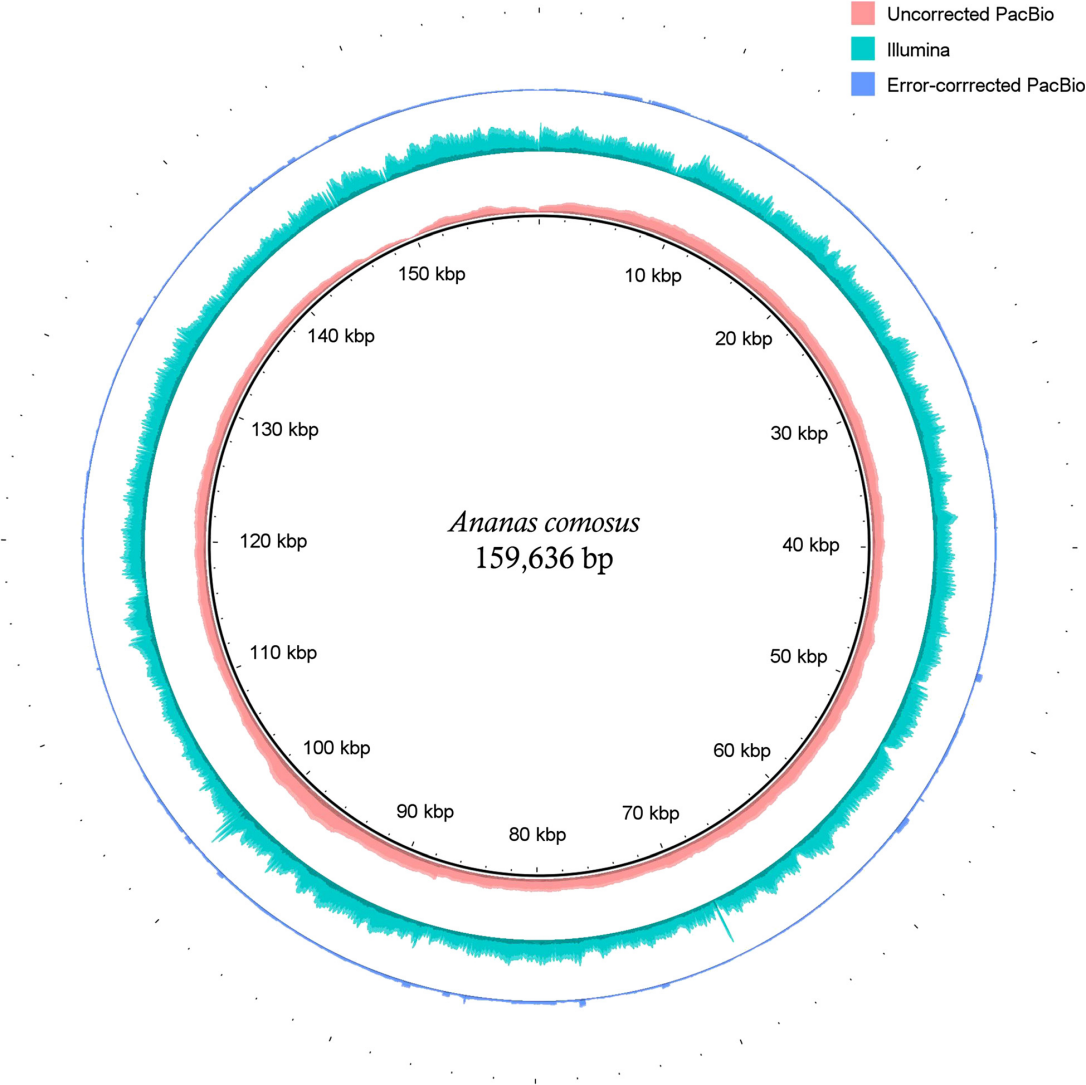
The coverage profile of the pineapple chloroplast after mapping back the error corrected, uncorrected and the Illumina short reads. Each three rings represented the depth of coverage from mapping back reads used in the assembly of the pineapple chloroplast genome. From the outermost to the innermost, the ring represents the corrected PacBio, the Illumina short reads and uncorrected PacBio mapped to the chloroplast genome of pineapple. The height of each ring is in proportion to the number of reads mapped across the chloroplast genome. Figure was illustrated using BRIG [41]

Blasr [34] was used to map back the corrected and uncorrected PacBio reads, and this gave average depth coverage of 544× and 142×, respectively (Fig. 2) and the alignment of the Illumina short reads onto the chloroplast was performed using Novoalign which showed an average depth of 1788×. Although the alignment of the short reads showed much higher average depth, the coverage fluctuated throughout the chloroplast. On the contrary, the PacBio reads coverage were more uniform with the exception of the IR region, where reads preferably mapped to one of the IR compared to the other (even when the –placeRepeatsRandomly blasr [34] parameter was used). Similar mapping behaviour of the Illumina reads as compared to PacBio reads was also observed in Ferrarini et al. [23].

Three regions in the *ndhB* gene that were corrected by Quiver, were selected for validation using PCR-based Sanger sequencing (Additional file 3). Sequence validation showed 100 % similarity. A single contig assembly of the pineapple’s chloroplast using error corrected PacBio reads is a direct evidence of its potential in *de novo* genome assembly. Previously, Ferrarini et al. [23] successfully assembled the chloroplast of *Potentilla micrantha* using error corrected PacBio reads which were assembled into 97 contigs by Celera and were eventually scaffolded into a single sequence using minimus2 and Seqman. In a separate study, Wu et al. [22] used lastz tools to assemble four Celera pre-assembled contigs into one final *Nelumbo nucifera* chloroplast. The pineapple chloroplast assembly by MIRA had successfully produced a single sequence without any additional scaffolding tools required. However, the orientation of the inverted repeat commonly present in the conserved chloroplast structure was misplaced and was not according to other quadripartite structure of published chloroplast genomes for land plants.

### Genome features

**Fig. 3:**
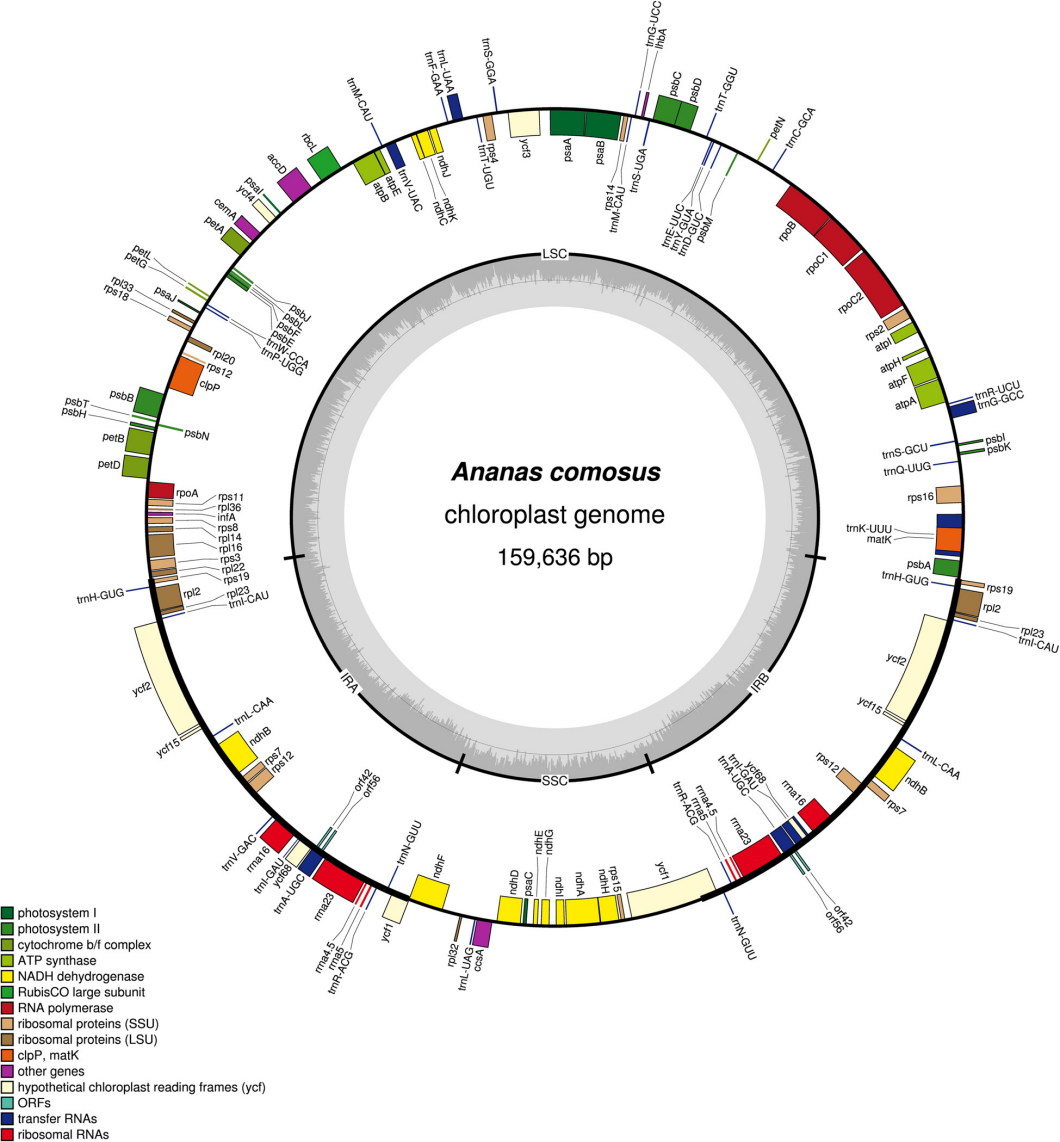
The *A. comosus* chloroplast genome. The figure shows circular representation of the pineapple chloroplast genome with structural organization of the gene content ring which was colour coded based on its functional category. The innermost circle denotes the GC content across the genome. The genes that were transcribed counter-clockwise and clockwise were at the outer and inner ring, respectively. The chloroplast illustration was drawn using OGDRAW [40]

The complete choloroplast genome of *A. comosus* was 159,636 bp in length (Genbank: KR336549). The complete pineapple chloroplast genome carried the conserved quadripartite structures usually found in land plants’ chloroplast genome. The genome consisted of a large single copy (LSC, 87,482 bp), inverted region A (IRA, 26,766 bp), short single copy (SSC, 18,622 bp) and inverted region B (IRB, 26,766 bp) (Fig. 3). Overall, the pineapple chloroplast genome had a GC content of 37.37 %. However, both the IRA and IRB regions had a higher GC content which was 42.74 % each, whereas the GC content for LSC and SSC were 35.36 % and 31.41 %, respectively. The gene content of the pineapple chloroplast was found to be most similar to that of *T. latifolia,* which is the sister clade of the Bromeliaceae family [49]. The genome consisted of 141 coding regions, with 117 unique regions and 24 regions which were repeated in the inverted region. Among these were 30 unique tRNAs with 8 duplications, 83 distinct peptide-coding genes; 12 were repeated and 4 rRNAs were all duplicated in the IR region (Table 1). As generally found in many other land plants, 18 genes were with introns, 12 were from protein-coding genes and 6 were from tRNAs. One gene, *rps12* was encoded as trans-spliced with a single 5′ end at the LSC region and a repeated 3′ end in both of the IR regions [50–52].

**Table 1:** List of genes in the chloroplast genome of pineapple

	Groups of genes	Name of genes
Protein synthesis and DNA-replication	Transfer RNAs	*trnC-GCA, trnD-GUC, trnE-UUC, trnF-GAA, trnfM-CAU, trnG-GCC, trnG-UCC, trnK-UUU, trnL-UAA, trnL-UAG, trnM-CAU, trnP-UGG, trnQ-UUG, trnR-UCU, trnS-GCU, trnS-GGA, trnS-UGA, trnT-GGU, trnT-UGU, trnV-UAC, trnW-CCA, trnY-GUA, trnA-UGC* (2×)*, trnH-GUG* (2×)*, trnI-CAU* (2×)*, trnI-GAU* (2×)*, trnL-CAA* (2×)*, trnN-GUU* (2×)*, trnR-ACG* (2×)*, trnV-GAC* (2×)
	Ribosomal RNAs	*rrn16* (2×)*, rrn23* (2×)*, rrn4.5* (2×)*, rrn5* (2×)
	Ribosomal protein small subunit	*rps16, rps2, rps14, rps4, rps18, rps12* (2×)*, rps11, rps8, rps3, rps19* (2×)*, rps7* (2×)*, rps15*
	Ribosomal protein large subunit	*rpl14, rpl16, rpl2* (2×)*, rpl20, rpl22, rpl23* (2×)*, rpl32, rpl33, rpl36*
	Subunits of RNA polymerase	*rpoA, rpoB, rpoC1, rpoC2*
Photosynthesis	Photosystem I	*psaA, psaB, psaC, psaI, psaJ*
	Photosystem II	*psbA, psbB, psbC, psbD, psbE, psbF, psbH, psbI, psbJ, psbK, psbL, psbM, psbN, psbT, lhbA*
	Cythochrome b/f complex	*petA, petB, petD, petG, petL, petN*
	ATP synthase	*atpA, atpB, atpE, atpF, atpH, atpI*
	NADH-dehydrogenase	*ndhA, ndhB* (2×)*, ndhC, ndhD, ndhE, ndhF, ndhG, ndhH, ndhI, ndhJ, ndhK*
	Large subunit Rubisco	*rbcL*
Miscellaneous group	Translation initiation factor IF-1	*infA*
	Acetyl-CoA carboxylase	*accD*
	Cytochrome c biogenesis	*ccsA*
	Maturase	*matK*
	ATP-dependent protease	*clpP*
	Inner membrane protein	*cemA*
Pseudogene unknown function	Conserved hypothetical chloroplast ORF	*ycf1* (2×)*, ycf15* (2×)*, ycf2* (2×)*, ycf3, ycf4, ycf68* (2×), *orf42* (2×), *orf56* (2×)

A single base change and five bases deletion occurred in pineapple chloroplast gene of *ycf15* which had caused a premature stop codon and frame-shift in the 3′ end region of the gene. A similar case was also observed in the *T. latifolia* chloroplast genome, even though this gene was unannotated in the current record [53] and also in other monocot chloroplast genomes such as *Phoenix dactylifera* (GenBank: NC013991.2) [54], and *D. bromeliifolius* (GenBank: NC020367.1). Altogether, the 95 protein-coding genes constituted a total of 82,389 bp including the repeated region and pseudogenes, and comprised of 27,361 codons, excluding the stop codons. Meanwhile, the most and the least prevalent amino acids coded were leucine (10.69 %) and cysteine (1.30 %), respectively (Table 2). This was similar to many other protein-coding genes in land plants’ chloroplast [55, 56].

**Table 2:** Relative synonymous codon usage (RSCU) for protein-coding genes in *Ananas comosus*

Codon	AA	ObsFreq	RSCU
UGA	*	26	0.772
UAG	*	31	0.921
UAA	*	44	1.307
GCU	A	630	1.819
GCG	A	151	0.436
GCC	A	210	0.606
GCA	A	394	1.138
UGU	C	246	1.46
UGC	C	91	0.54
GAU	D	914	1.616
GAC	D	217	0.384
GAG	E	414	0.563
GAA	E	1058	1.438
UUU	F	921	1.205
UUC	F	607	0.795
GGU	G	611	1.336
GGG	G	298	0.652
GGC	G	157	0.343
GGA	G	763	1.669
CAC	H	157	0.463
CAU	H	521	1.537
AUU	I	1120	1.431
AUA	I	715	0.914
AUC	I	513	0.655
AAA	K	1026	1.456
AAG	K	383	0.544
CUA	L	409	1.169
CUC	L	214	0.612
CUG	L	177	0.506
CUU	L	599	1.713
UUA	L	838	1.17
UUG	L	594	0.83
AUG	M	656	1
AAC	N	298	0.461
AAU	N	996	1.539
CCA	P	340	1.195
CCC	P	231	0.812
CCU	P	425	1.494
CCG	P	142	0.499
CAA	Q	721	1.488
CAG	Q	248	0.512
AGA	R	548	1.524
AGG	R	171	0.476
CGA	R	369	1.525
CGC	R	96	0.397
CGG	R	137	0.566
CGU	R	366	1.512
AGC	S	108	0.409
AGU	S	420	1.591
UCA	S	458	1.129
UCC	S	374	0.922
UCG	S	198	0.488
UCU	S	593	1.461
ACC	T	263	0.739
ACA	T	438	1.23
ACG	T	163	0.458
ACU	T	560	1.573
GUU	V	525	1.425
GUG	V	207	0.562
GUC	V	188	0.51
GUA	V	554	1.503
UGG	W	468	1
UAC	Y	218	0.424
UAU	Y	811	1.576

All of the tRNAs required for the protein synthesis of the protein coding genes of pineapple chloroplast were identified and their number and kinds were similar to that of other well-characterized chloroplast genomes. The RSCU analyses, showed that there was a strong bias to A or T at the third codon position as compared to G or C for all amino acids including the stop codons (Table 2). For example, the codon ending with either an A or T for the amino acid Alanine was 28 and 45 %, as compared to only 10 and 15 % for G or C, respectively. This phenomena was similarly observed in many other chloroplast genomes [57–59].

### Repeat analysis

A total of 35 repeat structures were identified in the chloroplast of pineapple genome, consisting of 3 dispersed, 8 palindromic and 24 forward tandem repeats with a minimum size of 30 bp (Table 3). The repeats were characterised based on Zhang et al. [60], where the forward repeat were assigned into either dispersed or tandem depending on the location of the repeating unit. The majority of the repeats were tandem and found in the LSC region (specifically in the intergenic spacer regions) while a few were in the coding sequences. Among coding regions that contained repeats were the *ycf2*, *psaB*, *psaA*, *rpoC2*, *ycf1* and *rps11*. Even though chloroplast genes are conserved across many land plants, repeats occurring in the coding regions may vary. For example, the coding sequences that contain repeats in *Camellia* cp genome are *infA*, *rps18*, *rps3* and *rpoC2* [61] whereas in *Datura stratomonium* cp genome, repeats in coding sequence occurred in *ndhF*, *ycf1*, *rps18*, *ycf2* and *psaA* [52].

**Table 3:** Repeat sequences for *Ananas comosus* chloroplast genome

No.	Type	Location	Region	Repeat unit	Period size (bp)	Copy number
1	T	trnS-GCU and trnG-GCC	LSC	TACATTAAACAATATTAAAT	20	2
2	D	psbI and trnG-GCC psbE and petL	LSC	TAAAAATATATATATATATATAAATATATTATAGTA	36	2
3	T	accD and psaI	LSC	TAATTAAGATAGACAA	16	2
4	T	accD and psaI	LSC	TTTTCATAAGAAAACTCCT	18	2
5	T	accD and psaI	LSC	ATTTGAGATTTCCAAATAATA	20	2
6	P	accD and psaI	LSC	GTATAATATGAAGTTTGAATAT	22	2
7	T	clpP (intron)	LSC	TTAGGACAAAATTGTATCTC	20	2
8	T	clpP (intron)	LSC	AGTAATAGTAGGTATAA	17	3
9	T	ndhB and trnL-CAA	IRA	GTCATTCAAGCGTAT	15	2
10	T	ndhC and trnV-UAC	LSC	ATTCTAAATAATAAAAG	17	2
11	T	ndhF and rpl32	SSC	TATTTATTAGATTTTGC	16	2
12	T	ndhF and rpl33	SSC	TCGGAAATCTTATGATACTCCTT	23	2
13	T	petD (intron)	LSC	TTATATGGGTTTATTTCTGTTA	22	2
14	P	petN and psbM	LSC	CTAAAGAGTGGTAGAAAGGACTA	24	2
15	D	psaB (CDS) and psaA (CDS)	LSC	TGCAATAGCTAAATGATGATGAGCAATATCGGTCA	34	2
16	P	psbA	LSC	AAAAAATACCCAATATCTTGT	21	2
17	P	psbT and psbH	LSC	ATTGAAGTAATGAGCCTCCCA	21	2
18	T	rbcL and accD	LSC	TATATACAAG	10	5
19	T	rpoC2 (CDS)	LSC	TGTCTCATGTAAATT	15	2
20	T	rps11 (CDS)	LSC	TACGCCCATTCTTACGTGAACCAA	24	2
21	P	trnD-GUC and trnE-UUC	LSC	TTTCATGATACTTTACTTA	19	2
22	T	trnF-GAA and ndhJ	LSC	TATTCTATTTCGTCA	15	2
23	T	trnL-CAA ndhB	IRB	ACATACGCTTGAATG	15	2
24	P	ycf1 (CDS)	SSC	TTTTATTTTGACTTGTATTTTTAT	22	2
25	T	ycf15 and trnL-CAA	IRB	GAATAACTAAAGAAAATAGATA	22	2
26	T	ycf15 and trnL-CAA	IRA	TCTATCTATTTTCTTTACTTAT	22	2
27	T	ycf2 (CDS)	IRB	TTTGTCCAAGTCACTTCTCTT	21	3
28	T	ycf2 (CDS)	IRB/IRA	CTTTTTGTCCAAGTCACTTCC	21	3
29	T	ycf2 (CDS)	IRB/IRA	GATATCGATATTGATGATAGTGAC	24	2
30	T	ycf2 (CDS)	IRA	GAAGTGACTTGGACAAAAAGA	21	3
31	D/P	ycf3 (intron) petB (intron) rps12 and trnV-GAC	LSC/IRA/IRB	CCAGAACCGTACATGAGATTTTCATCTCATACGGCTCCTC	39	3

*Letter T, D, and P in Region column represents Tandem, Dispersed and Palindromic repeats, respectively. IRA, IRB, LSC and SSC represents inverted region A, inverted region B, long single copy and short single copy, respectively. All of the repeat locations are in intergenic spacer regions, except otherwise indicated

One repeat, which was 39 bp in size and located in the intron of *ycf3* gene, was found to be repeated thrice, twice as palindromic repeats in the LSC and IRA regions, and once as a dispersed forward repeat in the IRB region. The largest repeat, with a size of 72 bp, was the dispersed repeat of intergenic spacer region of *psbI*/*trnG-GCC* and *psbE*/*petL* which constituted of a repeating unit of 36 bp in size occurring twice. Meanwhile, the *rpoC2* gene, which was known to be a highly divergent region in many chloroplast genomes [60, 62], was found to contain only a single repeat in the pineapple cp genome. In contrary, multiple chloroplast genomes from the grass family have shown to carry several sets of repeats in this coding region except for *Hordeum vulgare* [63].

### Simple Sequence Repeats (SSR)

Simple sequence repeats (SSR) or microsatellites are stretches of small repeating units of DNA occurring in both coding and non-coding regions. Due to its polymorphic nature and co-dominant mode of inheritance, these stretches of DNA have been used as DNA markers for population genetic studies and many more. In particular, chloroplast SSRs have been used ubiquitously to numerate genetic variations among plant genotypes [64–67]. In the pineapple chloroplast, 205 SSRs were identified *in-silico* using MISA, of which, 129 were mononucletotides, 59 were dinucleotides, 5 were trinucleotides and 12 were tetranucleotides. Fifty one SSRs occurred in compound formation that was made up of several combinations of SSRs interrupted by maximum distances of 100 bp [68]. The most abundant motifs were the runs of mononucleotide A/T, consisting about 61 %. The number was slightly lower than reported in previous studies on asterids (68 %) and monocots (76 %) [50, 69].

Among the pineapple SSRs, 48 were found in the coding region, with six genes harbouring at least two SSRs. These include *psbC, accD, cemA, petA, ycf2 and ycf1*. Even though chloroplasts contain conserved genes, the number of SSRs that they harbour and the coding sequences that contain the SSRs varies. For example, in the chloroplast of *D. stramonium*, only five genes (*atpA*, *ycf3*, *accD*, *rbcL* and *clpP*) contained SSRs [52] and these were different from the ones found in pineapple. The presence of SSRs in coding regions (Type II SSRs) raises concern as they are prone to mutation and any changes in the coding region may cause frame-shifts to occur and render the gene non-functional [70]. In comparison to the IR region, the SSRs were more prevalent in the LSC and SSC regions and this is coherent with other chloroplast genomes [56, 69, 71]. In addition, upon comparison with other commelinids, the LSC and SSC also contain regions with high sequence divergence to other chloroplast genomes. The complete list of the SSRs identified in the chloroplast of pineapple is given in Additional file 4.

### Chloroplast genomes comparison in the commelinids clade

**Fig. 4:**
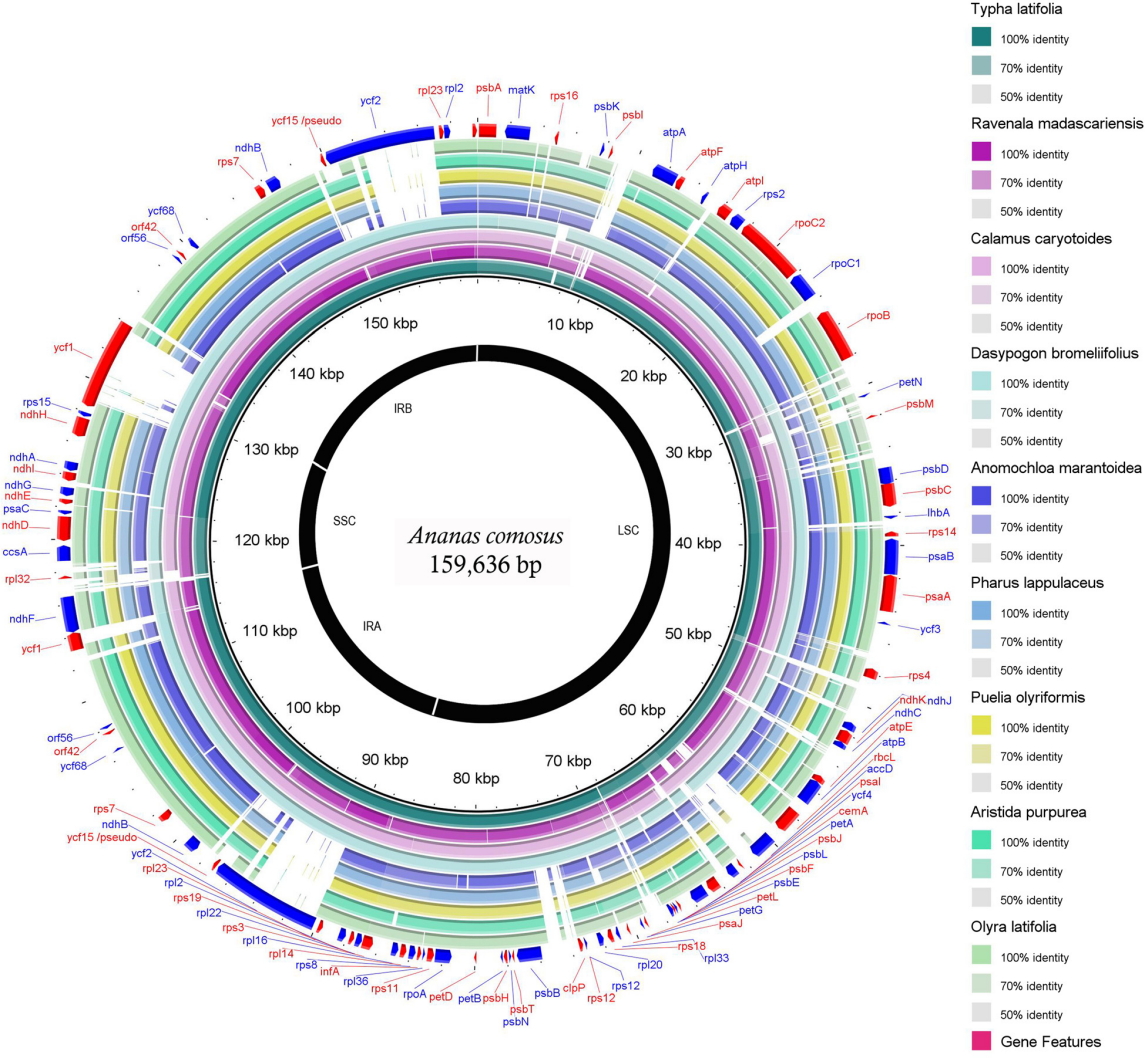
Genome comparison of nine chloroplast genomes from the subclass Commelinidaes to the pineapple chloroplast genome. From the third ring: *Typha latifolia* (green), *Ravenala madagascariensis* (purple), *Calamus caryotoides* (light purple), *Dasypogon bromelifolius* (turqoise), *Anomochloa marantoidea* (blue), *Pharus lappulaceus* (light blue)*, Puelia olyriformis* (yellow), *Aristida purpurea* (green) and *Olyra latifolia* (light green). Pairwise comparisons using blast n were performed on every chloroplast genome to the chloroplast genome of pineapple and produced alignments which were colour coded based on the similarity score: dark shade, lighter shade and grey depicts similarity score of above 90 %, above 80 % and below 80 %, respectively. The first outer rings are the protein-coding gene features positioned based on the pineapple chloroplast genome

Nine chloroplast genomes representing every order under the subclass Commelinidae were compared to the chloroplast of *A. comosus* (Fig. 4). The size of all the nine genomes ranged from 136,785 to 166,170 bp, with *O. latifolia* being the smallest and *R. madagascariensis* the largest in size, respectively. Compared to the *A. comosus* chloroplast genome (159,636 bp), all of the other Poales were much smaller by 22, 21, 19, 17 and 21 kb for *O. latifolia*, *A. purpurea*, *P. olyriformis*, *P. lappulaceus* and *A. marantoidea*, respectively. Similarly, *D. bromoliifolius* and *C. caryotoides* were also smaller by 1.7 and 2.3 kb than the pineapple chloroplast, respectively. Only the chloroplast genome of *T. latifolia* and *R. madagascariensis* were found to be bigger than the pineapple chloroplast by 1.9 and 6.5 kb in size, respectively.

In the analysis of sequence identity between the pineapple chloroplast and other representatives of the commelinids, the results showed consistent regions of the chloroplast genome that was either lost or diverged (sequence identity of less than 80 %). Previous record had shown that several member of the Poacea family had partial deletion of the *ycf1*, *accD* and *ycf2* gene [53] and this had caused their chloroplast genomes suffered much length reduction as compared to other members of commelinids clade. In relative comparison to pineapple chloroplast, the partial gene loss of *ycf1*, *accD* and *ycf2* gene extended to all member of the graminid clade (i.e. excluding *T.latifolia*). For all other members of the non-Poales, the region still maintained a homogeneity of above 90 % (i.e. indicated by the brightness of the ring’s colour in Fig. 4), with the exception of *R. madascariensis*. This species showed minimal deletions of the intergenic spacer at the same locations where gene loss occurred in graminid. However, the loss was minimal and all the three genes were still intact.

Overall, the region with the highest similarity across all nine chloroplast genomes in comparison to pineapple occurred in the IR region, specifically in between the genes *ycf1* and *ycf15*, with the exception of *D. bromoliifolius*. Similar with others, the prefix of the region of *D. bromoliifolius* had a high sequence similarity of above 90 %, and only when it reached the intergenic spacer *rps7*-*ycf68* the value dropped to below 90 %. Interestingly, where the sequence similarity changed for *D. bromoliifolius*, *A. marantoidea* had small deletion at that particular locus, but still maintained high homogeneity across this IR region. This evolutionary conserved feature of the IR region in chloroplast was reported in several other studies [72, 73] and Palmer [73] proposed that this is due to the conserved nature of the rRNA genes that are located only in the IR region. In this study, the conserved nature of the rRNA genes was also prominent among all members of the subclass of Commelinidae as shown at the region near to the genes *rpl2* and *rpl23*.

In parallel with other chloroplast genomes [74], the LSC and SSC regions endured much higher divergence. The least conserved region was in between the intergenic spacer of genes *rpoB* and *psb*D. This region had consistently low percent of identity to the *A. comosus* chloroplast genome in comparison across all the nine species. However, all of them still harboured the *petN* and *petM* genes contained in the region. Similar divergent pattern of the region between *rpoB* and *psbD* was also observed among four chloroplasts of duckweed [75]. On a different note, the region flanked by the *matK* and *atpA* genes also showed low similarity score throughout the region. This was prominent for all of the Poales family and *D. bromoliifolius* that showed sequence identity of below 80 % throughout the region, whereas the chloroplast of *C. caryotoides*, *R. madascariensis*, and *T. latofolia* still maintained similarity of above 80 % to the chloroplast of *A. comosus* but with several indels in intergenic spacer. It is apparent that the locations of the hotspot divergence across species in the commelinids were the same. Clearly, the pattern of divergence in the commelinids followed the same pattern across the order, especially in the intergenic spacer region, where gene loss was not detrimental to the organelle’s function. These highly divergent loci provide as an important clue for biologist to dissect the evolutionary changes in the chloroplast across taxa.

### Protein-coding gene sequence diversity of commelinid

In order to further investigate the divergent pattern of the pineapple chloroplast genome and other representatives of the commelinids, 80 protein-coding gene sequences were compared to derive the average pairwise sequence distance among the 100 members (Additional file 5). The results showed that a majority of the genes maintained low levels of average sequence distances. More than 92 % of the genes had a value of less than 0.1 and only six genes showed an average sequence distance value of more than 0.1. The six genes were *ycf1*, *accD*, *rpl32*, *rpl22*, *matK* and *infA*, arranged from the most to the least divergent. All of these genes were found located in the single copy region either in the LSC or SSC as indicated in the table. The six genes were also cited to be among the ten most divergent genes in the asterids clade [50] and five of them (except *rpl32*) were recorded as the most divergent among 17 vascular plants and *Panax* sp [56]. Nie et al. [57] suggested that the high average sequence distance of gene *ycf1* was mainly due to the occurance of various indels among the members of the asterids clade. From the deletion/insertion polymorphisms (DIPs) analysis of the aligned *ycf1* gene, 197 indel events were recorded among the 100 taxa. The other five most diverged genes only contained indel event of less than 31 (data not shown). Even though *ycf1* has been lost among the graminids, and it contains many indels which may render the gene non-functional, the gene has been proven essential in tobacco [76]. Majority of genes involved in photosystem I or II had low sequence divergence of below 0.05 with the exception for *psbK, pshH* and *psbM*. This indicated that the chloroplasts maintained relatively low rate of sequence divergence to preserve its primary function. Other parasitic plants that have lost the photosynthetic capability have shown to have lost the functional photosynthetic genes [77, 78]. However, it is incorrect to assume that any genes with high level of sequence divergence will soon lose its function, turning into pseudogenes. For example, *matK* is known to have high sequence divergence not only in commelinids but also in many other clades. In spite of its level of divergence, the product of *matK* is crucial as it is the only group II intron maturase available in chloroplast to perform RNA splicing in intron-containing genes [79]. This gene somehow is able to accommodate the high substitution rate but still maintain conserved secondary structure that is important for its function as maturase [80]. Meanwhile, *petN* and *psbM* genes are located in regions where it most diverged among the nine chloroplast genome in relative comparison to the pineapple cp genome. However, both of these genes had relatively low sequence distance of 0.008 and 0.015, respectively. This indicated that in spite of the many occurrences of rearrangements surrounding the genes, the organelle has still maintained the intactness of the gene that was deemed important.

### Selection pressure on *A. comosus* chloroplast genome

The non-synonymous (Ka) to synonymous rate ratio (denoted by Ka/Ks) were used to assess the rate of divergence between gene sequences and in turn to determine its relative effect of positive, neutral or purifying selection. Ka/Ks ratio of more than one indicates positive selection, while a value of less than one indicates purifying selection. A value of 0 indicates the presence of neutral selection. The ratio has been used in many studies to detect the evolutionary forces being imposed on certain set of genes [81–83].

**Fig. 5:**
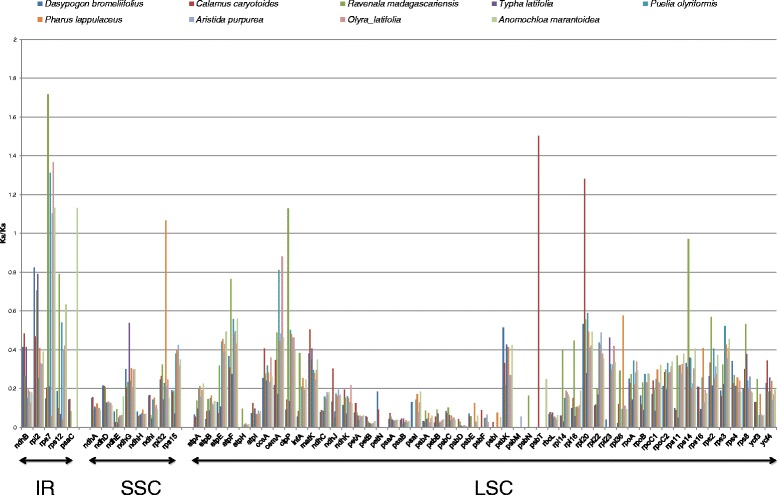
The Ka/Ks ratio of the protein-coding gene from the nine members of commelinid for comparison with *A. comosus*

In this study, the Ka/Ks ratio was calculated for 64 protein-coding genes in common across all nine chloroplast genomes. The result is summarized in Fig. 5. When computing the values, there were genes with Ka/Ks values of 50 and NA. This happened when the Ks was extremely low or when there were no substitution in the alignment (i.e. 100 % match), respectively. For both cases, the values of 50 and NA were changed to 0. Overall, the ratio of Ka/Ks of protein-coding genes throughout the chloroplast was higher in genes within the IR region and lower for genes in the SSC region. However, the lowest Ka/Ks value was observed for genes involved in the cytochrome b/f complex biogenesis (*petA*, *petB*, and *petN*), photosystem I (*psaA* and *psaB*) and photosystem II (PSII) (*psbA, psbB, psbC, psbD, psbE, psbF, psbI, psbM* and *psbT* (with the exception of *C. caryotoides*)). This concurs correctly with the Kimura-corrected average pairwise distances for all the 100 chloroplast of the commelinids (as discussed the above section) as essential genes vital to the chloroplast function had the lowest level of genetic divergence. Among the genes involved in photosystem II only *psbK* had high Ka/Ks ratio as compared to gene members in the same functional group. In functional study of the gene, it was shown that the *psbK* gene was not directly required for the function of PSII but is necessary to associate other genes important for the stabilization of the thylakoid membrane [84].

The highest Ka/Ks value among the protein-coding genes across the nine chloroplast genomes was *rps7* despite of its low average pairwise sequence divergence (K2P) value. Five out of the nine genomes compared to pineapple showed Ka/Ks values of more than one, which indicates positive selection exerted on the genes in the pineapple chloroplast. To investigate further, the calculation of Ka/Ks ratio was extended to all 100 commelinids used in this phylogenetic study (Additional file 6) and to simplify the calculation for 100 sequences of *rps7* gene, the calculation was performed using Codon-based Z test of selection to test the hypothesis of positive selection (null hypothesis: strict-neutrality (dN = dS)) (dN/dS is equivalent to Ka/Ks). The result showed 12 species with significant higher number of non-synonymous substitution (denoted by *d*_*N*_) at P less than 0.05. These species were *Triticum aestivum*, *Olyra latifolia*, *Oryza sativa* Japonica, *O. australiensis*, *O. glaberrima*, *O. meridionalis*, *O. nivira*, *Leersia tisseranti, Anomochloa merantoidea*, *Danthonia california*, *Ravenala madagascariensis*, and *Rhynchoryza subulata*. In brief, the *rps7* gene product encodes for a small ribosomal protein that is crucial for the assembly and stability of ribosome. The gene is not limited to chloroplast but can also be found in nuclear and mitochondrial genomes. The specific function of this gene in chloroplast is still limited but efforts to understand its translation and its protein interaction has supported its function as a translation initiator in chloroplast [85–87]. In many comparative studies of chloroplast protein coding genes, the distance value for *rps7* gene sequence was mainly small indicating low levels of sequence divergence [88, 89]. In spite of that, high *d*_N_/*d*_S_ value for the gene was also reported among the Poacea in [53] which is similar to our findings. The result provides an indication of the selective pressure imposed on *rps7* in the chloroplast of pineapple. It serves as a clue that in the overall genome conservation of other genes, a single gene showed significantly higher number of non-synonymous substitution as compared to the other 12 species under the commelinids.

### IR contraction and expansion

**Fig. 6:**
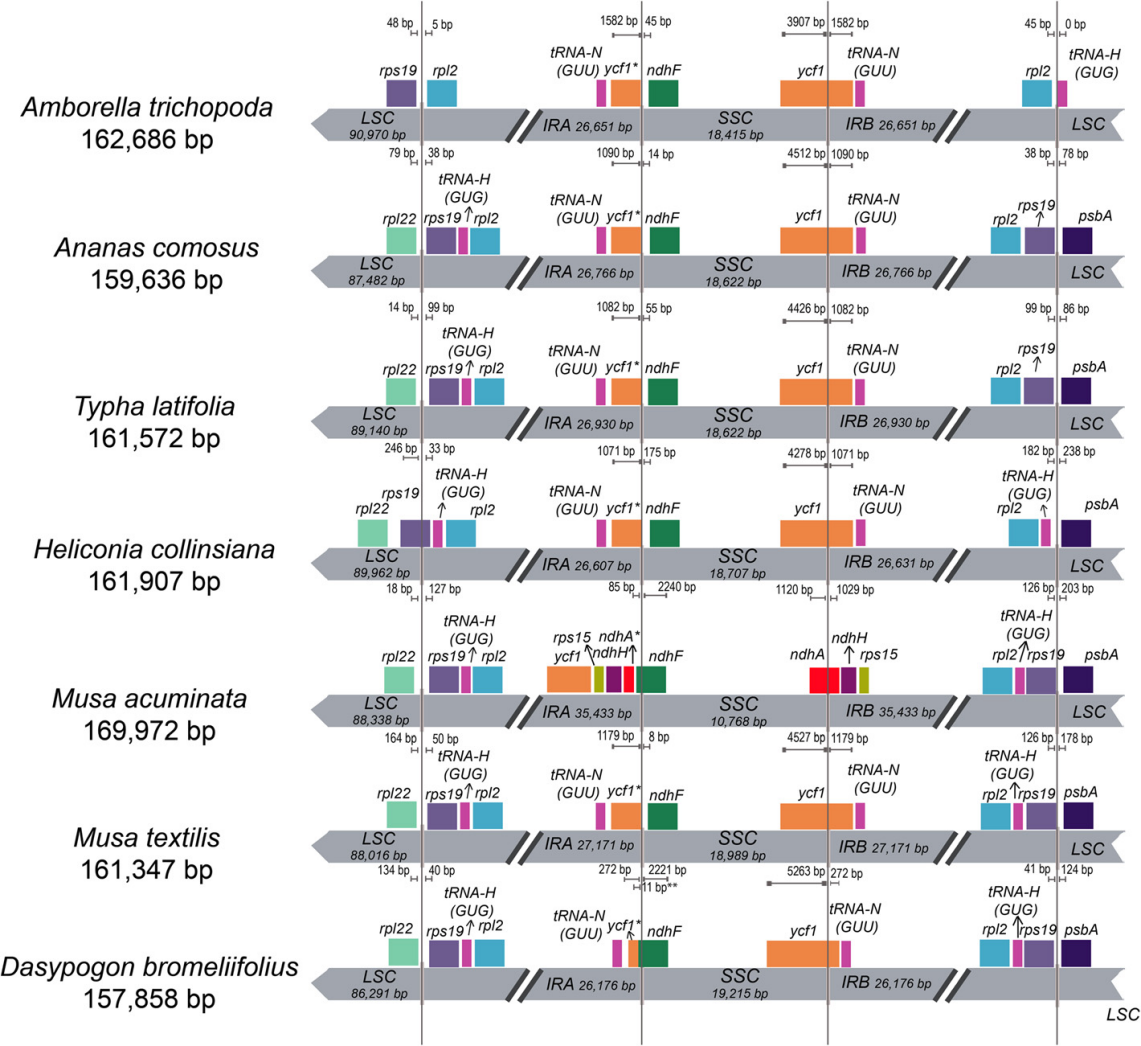
Comparison of chloroplast borders of LSC, SSC and IR regions among the species from subclass Commelinidae

Many evidences have shown that *Amborella trichopoda* is the only surviving sister lineage to angiosperms [90]. Even though some have refuted its position at the basal position of the angiosperm [91], the species still serves as a universal standard in the study of the structural variation since the divergence of angiosperm. In this study, the same nine chloroplast genomes representing the subclass Commelinidae were compared to the chloroplast of *A. trichopoda* to study the expansion of the inverted repeats region border (Fig. 6). In comparison to the *A. trichopoda*, the chloroplast sequence of *A. comosus* and *T. latifolia* did not show any major expansion except for the inclusion of the *rps19* gene and *trnH-GUG* tRNA at the IRA/LSC border. Similar observation was also made of the other members of commelinids in Martin et al. [92]. There was no unique expansion or contraction of the IRA region for *D. bromeliifolius*, which is the basal most of the commelinids clade (based on [29] and as observed in this study). However, the partial inclusion of the *ycf1* gene into the IRB region was only 285 bp in size, making it significantly lesser when compared to *T. latifolius* and *A. comosus* which included 1082 and 1146 bp of the *ycf1* gene into the same region, respectively.

In the chloroplast of banana (*Musa acuminata*), a major expansion of IR was reported [92] and the IR extended to include three additional genes (*rps15, ndhH* and *ycf1*) and a partial sequence of *ndhA*. Such extension was not observed in any other monocot even within its same genus, *Musa textilis* which only had an extension similar to what was observed in *A. comosus*. Similarly, its sister taxa, *Heliconia collinsiana* (in the same Zingiberales family) also had no major extension at the IR/SSC border. The major extension seen in *M. accuminata* seemed to be exclusive only to the species. However, to a certain extent the grass family also showed moderate extension of the IR border to include *ycf1* and *rps15* genes and partial sequences of *ndhH* gene as reported elsewhere [63, 93, 94]. Generally, multiple events of the IR expansions were observed among the angiosperms and these events were believed to have occurred in multiple steps and independently of each other [92, 95]. Overall, it can be deduced that the expansion and contraction of IR region in *A. comosus* and other commelinids is still stable since the divergence of angiosperm, with the exception to a single species of *Zingiberales*.

### Phylogenetic analysis

**Fig. 7:**
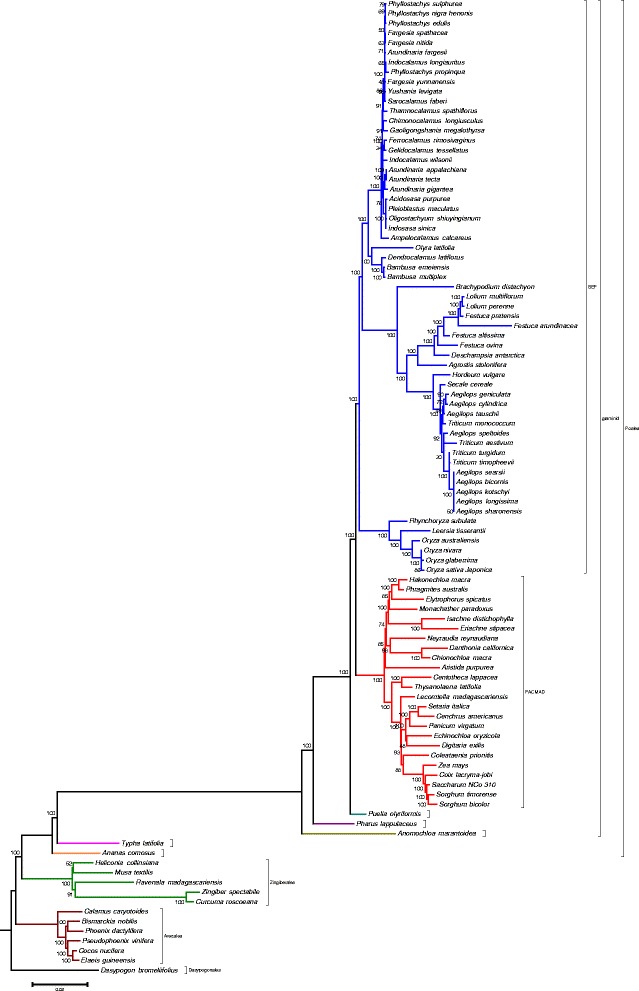
Phylogenetic tree of all available commelinids’ complete chloroplast sequences including the newly sequenced *A. comosus* chloroplast, in total of 100 taxa. The analysis was inferred using 56 protein coding sequences by maximum likelihood analysis with –lnL of 400419.797049 and bootstrap of 1000 replicates values were shown at the nodes

Fifty-six protein coding genes common to all 100 commelinids chloroplast from the NCBI Organelle Genome Resources including the pineapple chloroplast were used to infer its phylogeny. The maximum likelihood (ML) analysis by RAxML produced a tree with –lnL of 400419.797049. Bootstrap analysis with 1000 replications showed that 55 out of 100 nodes had 100 % values, with only 5 nodes having values below 50 % (Fig. 7). The tree obtained suggested a correct phylogeny inference as the topology of the tree followed that of the latest Angiosperm Phylogeny Group III [9]. *A. comosus*, the only representative for Bromeliaceae, was formed as the earliest divergent clade leading to the split of the Poales from other members of commelinids. The tree also supports the phylogeny of Arecales as monophyletic to Dasypogonaceae and, Zingiberales was monophyletic to the Poales with 100 % bootstrap value as described by Barret et al. [29]. In this study only 56 protein-coding genes common to all 100 taxa were used in the analysis, in comparison to 83 set of protein coding genes in [29]. Despite of the lower number of gene set used in the analysis, the tree produced were able to validate the relationship between the family of the subclass commelinid obtained in previous study with robust support. In the tree, the branch length for *T. latifolia* was longer than *A. comosus*, indicating a higher rate of change in *T. latifolia*. This serves as an indication that the Bromeliaceae family as represented by pineapple to be at the basal most of the Poales order. In congruent with other studies, the Poacea family showed longer branch as it diverged from other Poales members but with shorter internal branch [29, 49]. The long branch of the Poales indicated that the graminids in Poales experienced faster rates of plastid sequence evolution as compared to other members of subclass Commelinidae.

## Conclusions

We successfully assembled, annotated and analysed the complete chloroplast sequence of the MD-2 hybrid pineapple (*A. comosus*). The chloroplast genome serves as a valuable example of how the presence of large repeats can cause assembly error and would impose a considerable challenge to the *de novo* assembly of a genome. Long reads, similar to that produced by PacBio would help in the assembly of complex genomes, as long as the reads are longer than the repeats. However, with the limitation of the error rate, a required error correction module would reduce the length of the long sequence reads and hence would limit the potential of the sequence read to resolve long repeats. Perhaps, a new algorithm for error correction could mediate the reduced length problem faced in this study. In relative to the earliest angiosperm species *A. trichopoda,* the pineapple chloroplast genome is still highly conserved and is found to be very similar to its sister taxon, *T. latifolia*. Analysis of 56 chloroplast genes datasets confirmed the phylogeny of the commelinids as *Arecales*, *Dasypogonaceae*, *Zingiberales, Commelinales* and *Poales* with robust support. The availability of the pineapple chloroplast genome will serve as a valuable reference for the comparative studies of the Bromeliaceae to other monocots or angiosperms and most importantly it will make other molecular biology applications such as chloroplast gene transformation feasible.

### Availability of supporting data

The complete chloroplast genome of MD-2 pineapple has been submitted to the GenBank (accession KR336549). The nucleic acid matrices and tree for phylogenetic analysis can be found in TreeBASE website at this link http://purl.org/phylo/treebase/phylows/study/TB2:S18029?x-accesscode=2a8e85af14b2eb2378b3d6c838f1fa90&format=html. Other data used in the analysis are included within the article and its additional files.

## Additional files

Additional file 1:
**Accession number of chloroplast from green plants. Accession number of chloroplast from green plants (Viridiplantae) used to bait chloroplast reads from total pineapple genomic DNA PacBio sequencing read.** (DOCX 35 kb) Additional file 2:
**The final nucleic acid alignment used in phylogenetic analysis. Eighty genes from all the available chloroplasts from the subclass commelinid were aligned using MAFFT aligner tools and were used to infer its phylogeny.** The file provided is in FASTA format. (TXT 2634 kb) Additional file 3:
**PCR validation of Quiver correction on the chloroplast genome of pineapple.** Three regions corrected by the Quiver tool using PacBio sequences reads were validated using PCR-based sequencing. The alignment file of the validation read sequence onto the chloroplast is provided in the FASTA format. The dash represents the gap between the Sanger sequence reads to the reference *ndhB* gene. (TXT 6 kb) Additional file 4:
**Complete list of the SSRs identified in the chloroplast of pineapple.** SSRs identified using Misa in *A. comosus* chloroplast genome. (DOCX 38 kb) Additional file 5:
**Estimates of average evolutionary divergence over 80 protein coding-gene sequences from the subclass commelinid.** Standard error estimate(s) are shown in the second column and were obtained by a bootstrap procedure (1000 replicates). Analyses were conducted using the Kimura 2-parameter model [1]. The analysis involved 100 nucleotide sequences. Codon positions included were 1st + 2nd + 3rd + Noncoding. All positions with less than 95 % site coverage were eliminated. That is, fewer than 5 % alignment gaps, missing data, and ambiguous bases were allowed at any position. There were a total of 465 positions in the final dataset. Evolutionary analyses were conducted in MEGA6. (DOCX 29 kb) Additional file 6:
**Codon-based Z test of positive selection for** ***rps7*** **gene from 100 species of subclass commelinid including the** ***A. comosus***. The probability of accepting alternative hypothesis of positive selection (dN > dS) is shown (below diagonal). Values of P less than 0.05 are considered significant at the 5 % level and are highlighted. The test statistic (dN - dS) is shown above the diagonal. dS and dN are the numbers of synonymous and nonsynonymous substitutions per site, respectively. The variance of the difference was computed using the analytical method. Analyses were conducted using the Nei-Gojobori method. The analysis involved 100 nucleotide sequences. All ambiguous positions were removed for each sequence pair. There were a total of 196 positions in the final dataset. Evolutionary analyses were conducted in MEGA6. (XLS 166 kb)

## Abbreviations

cp: Chloroplast
LSC: Large single copy
SSC: Small single copy
IR: Inverted repeat
IRA: Inverted repeat A
IRB: Inverted repeat B
RSCU: Relative synonymous codon usage
SSR: Simple sequence repeat
NGS: Next generation sequencing
