# Erratum to: Complete chloroplast genome sequence of MD-2 pineapple and its comparative analysis among nine other plants from the subclass Commelinidae

**DOI:** 10.1186/s12870-015-0619-x

**Published:** 2015-12-18

**Authors:** RM Redwan, A. Saidin, SV Kumar

**Affiliations:** Biotechnology Research Institute, Universiti Malaysia Sabah, Jalan UMS, 88400 Kota Kinabalu, Sabah Malaysia; Novocraft Technology Sdn. Bhd, Two Square, Seksyen 19, Petaling Jaya, Selangor Malaysia

Erratum

After the publication of this work [1], we noticed that an incorrect version of Fig. 2 (Fig. 1 here) was published. The correct version of Fig. 2 has been corrected in the original article and is also included correctly below. The publisher apologizes for any inconvenience caused.

**Fig. 1:**
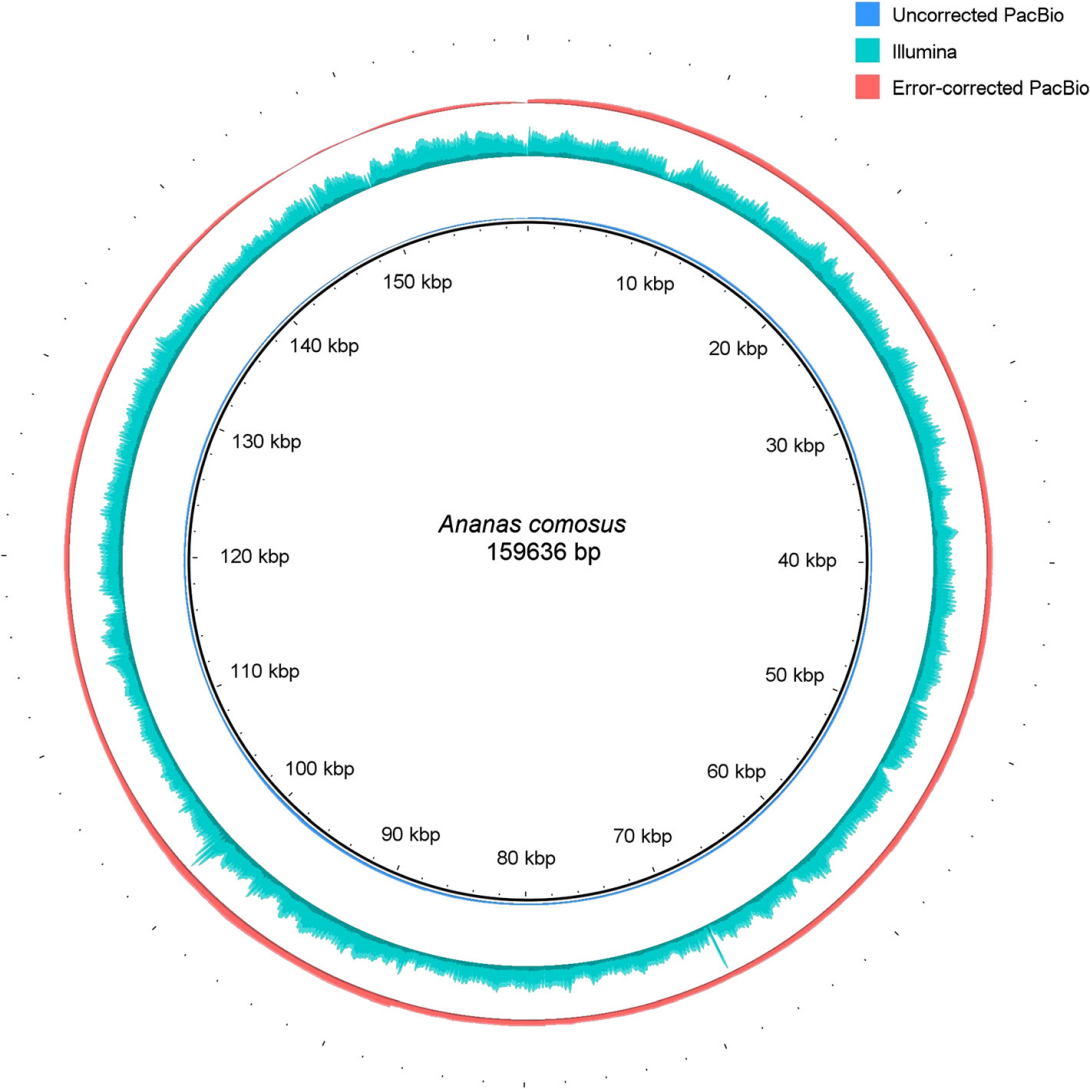
The coverage profile of the pineapple chloroplast after mapping back the error corrected, uncorrected and the Illumina short reads. Each three rings represented the depth of coverage from mapping back reads used in the assembly of the pineapple chloroplast genome. From the outermost to the innermost, the ring represents the corrected PacBio, the Illumina short reads and uncorrected PacBio mapped to the chloroplast genome of pineapple. The height of each ring is in proportion to the number of reads mapped across the chloroplast genome. Figure was illustrated using BRIG [41]
