# Supplementary material for: Complete chloroplast genome sequence of MD-2 pineapple and its comparative analysis among nine other plants from the subclass Commelinidae
Source: BMC Plant Biol. 2015 Aug 12;15:196. doi: 10.1186/s12870-015-0587-1 (PMC4534033; doi:10.1186/s12870-015-0587-1)
Supplement: Additional file 1: — Accession number of chloroplast from green plants. Accession number of chloroplast from green plants (Viridiplantae) used to bait chloroplast reads from total pineapple genomic DNA PacBio sequencing read. (DOCX 35 kb) [file 12870_2015_587_MOESM1_ESM.docx]

Accession number of chloroplast from green plants (Viridiplantae) used to bait chloroplast reads from total pineapple genomic DNA PacBio sequencing read.

| NCBI Accession No. | Description |
| --- | --- |
| NC_001631.1 | Pinus thunbergii chloroplast, complete genome |
| NC_001865.1 | Chlorella vulgaris chloroplast, complete genome |
| NC_000932.1 | Arabidopsis thaliana chloroplast, complete genome |
| NC_002186.1 | Mesostigma viride chloroplast, complete genome |
| NC_001319.1 | Marchantia polymorpha chloroplast, complete genome |
| NC_001320.1 | Oryza sativa Japonica Group plastid, complete genome |
| NC_001568.1 | Epifagus virginiana chloroplast, complete genome |
| NC_000927.1 | Nephroselmis olivacea chloroplast, complete genome |
| NC_002202.1 | Spinacia oleracea plastid, complete genome |
| NC_001666.2 | Zea mays chloroplast, complete genome |
| NC_002694.1 | Lotus japonicus chloroplast, complete genome |
| NC_002762.1 | Triticum aestivum chloroplast, complete genome |
| NC_003119.6 | Medicago truncatula chloroplast, complete genome |
| NC_003386.1 | Psilotum nudum chloroplast, complete genome |
| NC_004115.1 | Chaetosphaeridium globosum chloroplast, complete genome |
| NC_004543.1 | Anthoceros formosae chloroplast, complete genome |
| NC_004561.1 | Atropa belladonna chloroplast, complete genome |
| NC_004766.1 | Adiantum capillus-veneris chloroplast, complete genome |
| NC_004993.1 | Calycanthus floridus var. glaucus chloroplast, complete genome |
| NC_005086.1 | Amborella trichopoda chloroplast, complete genome |
| NC_005087.1 | Physcomitrella patens subsp. patens chloroplast, complete genome |
| NC_005353.1 | Chlamydomonas reinhardtii chloroplast, complete genome |
| NC_005878.2 | Saccharum hybrid cultivar SP-80-3280 chloroplast, complete genome |
| NC_005973.1 | Oryza nivara chloroplast, complete genome |
| NC_006050.1 | Nymphaea alba chloroplast, complete genome |
| NC_006084.1 | Saccharum hybrid cultivar NCo 310 chloroplast, complete genome |
| NC_006290.1 | Panax ginseng chloroplast, complete genome |
| NC_006861.1 | Huperzia lucidula chloroplast, complete genome |
| NC_007144.1 | Cucumis sativus chloroplast, complete genome |
| NC_007407.1 | Acorus calamus plastid, complete genome |
| NC_007500.1 | Nicotiana sylvestris chloroplast, complete genome |
| NC_007499.1 | Phalaenopsis aphrodite subsp. formosana chloroplast, complete genome |
| NC_007578.1 | Lactuca sativa chloroplast, complete genome |
| NC_001879.2 | Nicotiana tabacum plastid, complete genome |
| NC_007602.1 | Nicotiana tomentosiformis chloroplast, complete genome |
| NC_007944.1 | Gossypium hirsutum chloroplast, complete genome |
| NC_007943.1 | Solanum bulbocastanum chloroplast, complete genome |
| NC_007942.1 | Glycine max chloroplast, complete genome |
| NC_007957.1 | Vitis vinifera chloroplast, complete genome |
| NC_007977.1 | Helianthus annuus chloroplast, complete genome |
| NC_008101.1 | Scenedesmus obliquus chloroplast, complete genome |
| NC_008097.1 | Chara vulgaris chloroplast, complete genome |
| NC_008099.1 | Oltmannsiellopsis viridis chloroplast, complete genome |
| NC_008100.1 | Helicosporidium sp. ex Simulium jonesii plastid, complete genome |
| NC_008117.1 | Zygnema circumcarinatum chloroplast, complete genome |
| NC_008116.1 | Staurastrum punctulatum chloroplast, complete genome |
| NC_008114.1 | Pseudendoclonium akinetum chloroplast, complete genome |
| NC_008115.1 | Eucalyptus globulus subsp. globulus chloroplast, complete genome |
| NC_008155.1 | Oryza sativa Indica Group chloroplast, complete genome |
| NC_008235.1 | Populus alba chloroplast, complete genome |
| NC_008289.1 | Ostreococcus tauri chloroplast, complete genome |
| NC_008326.1 | Liriodendron tulipifera chloroplast, complete genome |
| NC_008325.1 | Daucus carota chloroplast, complete genome |
| NC_008334.1 | Citrus sinensis chloroplast, complete genome |
| NC_008335.1 | Platanus occidentalis chloroplast, complete genome |
| NC_008336.1 | Nandina domestica chloroplast, complete genome |
| NC_008359.1 | Morus indica chloroplast, complete genome |
| NC_008372.1 | Stigeoclonium helveticum chloroplast, complete genome |
| NC_008407.1 | Jasminum nudiflorum chloroplast, complete genome |
| NC_008454.1 | Pelargonium x hortorum chloroplast, complete genome |
| NC_008456.1 | Drimys granadensis chloroplast, complete genome |
| NC_008457.1 | Piper cenocladum chloroplast, complete genome |
| NC_008535.1 | Coffea arabica chloroplast, complete genome |
| NC_008591.1 | Agrostis stolonifera chloroplast, complete genome |
| NC_008590.1 | Hordeum vulgare subsp. vulgare chloroplast, complete genome |
| NC_008602.1 | Sorghum bicolor chloroplast, complete genome |
| NC_008641.1 | Gossypium barbadense chloroplast, complete genome |
| NC_008788.1 | Nuphar advena chloroplast, complete genome |
| NC_008796.1 | Ranunculus macranthus chloroplast, complete genome |
| NC_008822.1 | Chlorokybus atmophyticus chloroplast, complete genome |
| NC_008829.1 | Angiopteris evecta chloroplast, complete genome |
| NC_009143.1 | Populus trichocarpa chloroplast, complete genome |
| NC_009270.1 | Capsella bursa-pastoris chloroplast, complete genome |
| NC_009259.1 | Phaseolus vulgaris chloroplast, complete genome |
| NC_009269.1 | Barbarea verna chloroplast, complete genome |
| NC_009273.1 | Lepidium virginicum chloroplast, complete genome |
| NC_009275.1 | Nasturtium officinale chloroplast, complete genome |
| NC_009266.1 | Aethionema grandiflorum chloroplast, complete genome |
| NC_009267.1 | Olimarabidopsis pumila chloroplast, complete genome |
| NC_009268.1 | Arabis hirsuta chloroplast, complete genome |
| NC_009271.1 | Crucihimalaya wallichii chloroplast, complete genome |
| NC_009272.1 | Draba nemorosa chloroplast, complete genome |
| NC_009274.1 | Lobularia maritima chloroplast, complete genome |
| NC_009265.1 | Aethionema cordifolium chloroplast, complete genome |
| NC_004677.2 | Pinus koraiensis chloroplast, complete genome |
| NC_009598.1 | Chloranthus spicatus chloroplast, complete genome |
| NC_009601.1 | Dioscorea elephantipes chloroplast, complete genome |
| NC_009600.1 | Illicium oligandrum chloroplast, complete genome |
| NC_009599.1 | Buxus microphylla chloroplast, complete genome |
| NC_009618.1 | Cycas taitungensis chloroplast, complete genome |
| NC_009681.1 | Leptosira terrestris chloroplast, complete genome |
| NC_009766.1 | Cuscuta reflexa chloroplast, complete genome |
| NC_009765.1 | Cuscuta gronovii chloroplast, complete genome |
| NC_009808.1 | Ipomoea purpurea chloroplast, complete genome |
| NC_009949.1 | Cuscuta obtusiflora chloroplast, complete genome |
| NC_009950.1 | Lolium perenne chloroplast, complete genome |
| NC_009962.1 | Ceratophyllum demersum chloroplast, complete genome |
| NC_009963.1 | Cuscuta exaltata chloroplast, complete genome |
| NC_010093.1 | Acorus americanus chloroplast, complete genome |
| NC_010109.1 | Lemna minor chloroplast, complete genome |
| NC_002693.2 | Oenothera elata subsp. hookeri plastid plastome I, complete sequence |
| NC_010323.1 | Carica papaya chloroplast, complete genome |
| NC_010358.1 | Oenothera argillicola chloroplast, complete genome |
| NC_010359.1 | Aneura mirabilis chloroplast, complete genome |
| NC_010360.1 | Oenothera glazioviana chloroplast, complete genome |
| NC_010361.1 | Oenothera biennis chloroplast, complete genome |
| NC_010362.1 | Oenothera parviflora chloroplast, complete genome |
| NC_010433.1 | Manihot esculenta chloroplast, complete genome |
| NC_010442.1 | Trachelium caeruleum chloroplast, complete genome |
| NC_010548.1 | Cryptomeria japonica chloroplast, complete genome |
| NC_010601.1 | Guizotia abyssinica chloroplast, complete genome |
| NC_010654.1 | Welwitschia mirabilis chloroplast, complete genome |
| NC_010776.1 | Fagopyrum esculentum subsp. ancestrale chloroplast, complete genome |
| NC_011032.1 | Brachypodium distachyon chloroplast, complete genome |
| NC_011031.1 | Oedogonium cardiacum chloroplast, complete genome |
| NC_011163.1 | Cicer arietinum chloroplast, complete genome |
| NC_011828.1 | Trifolium subterraneum chloroplast, complete genome |
| NC_011930.1 | Keteleeria davidiana chloroplast, complete genome |
| NC_011942.1 | Gnetum parvifolium chloroplast, complete genome |
| NC_011954.1 | Ephedra equisetina chloroplast, complete genome |
| NC_012052.1 | Syntrichia ruralis chloroplast, complete genome |
| NC_012099.1 | Pyramimonas parkeae chloroplast, complete genome |
| NC_012097.1 | Pycnococcus provasolii chloroplast, complete genome |
| NC_012101.1 | Monomastix sp. OKE-1 chloroplast, complete genome |
| NC_012224.1 | Jatropha curcas chloroplast, complete genome |
| NC_012575.1 | Micromonas sp. RCC299 chloroplast, complete genome |
| NC_012615.1 | Megaleranthis saniculifolia chloroplast, complete genome |
| NC_011152.3 | Picea sitchensis chloroplast, complete genome |
| NC_012818.1 | Alsophila spinulosa chloroplast, complete genome |
| NC_012927.1 | Bambusa oldhamii chloroplast, complete genome |
| NC_012978.1 | Parachlorella kessleri chloroplast, complete genome |
| NC_011713.2 | Festuca arundinacea chloroplast, complete genome |
| NC_013086.1 | Selaginella moellendorffii plastid, complete genome |
| NC_013088.1 | Dendrocalamus latiflorus chloroplast, complete genome |
| NC_013273.1 | Coix lacryma-jobi chloroplast, complete genome |
| NC_013359.1 | Bryopsis hypnoides chloroplast, complete genome |
| NC_013553.1 | Parthenium argentatum chloroplast, complete genome |
| NC_013823.1 | Typha latifolia chloroplast, complete genome |
| NC_013843.1 | Vigna radiata chloroplast, complete genome |
| NC_008096.2 | Solanum tuberosum chloroplast, complete genome |
| NC_014062.1 | Anomochloa marantoidea chloroplast, complete genome |
| NC_014063.1 | Lathyrus sativus chloroplast, complete genome |
| NC_014057.1 | Pisum sativum chloroplast, complete genome |
| NC_014056.1 | Oncidium Gower Ramsey chloroplast, complete genome |
| NC_013991.2 | Phoenix dactylifera chloroplast, complete genome |
| NC_014348.1 | Pteridium aquilinum subsp. aquilinum chloroplast, complete genome |
| NC_014346.1 | Floydiella terrestris chloroplast, complete genome |
| NC_014592.1 | Cheilanthes lindheimeri chloroplast, complete genome |
| NC_014569.1 | Erodium texanum plastid, complete genome |
| NC_014573.1 | Geranium palmatum plastid, complete genome |
| NC_014582.1 | Monsonia speciosa plastid, complete genome |
| NC_014575.1 | Cedrus deodara chloroplast, complete genome |
| NC_014589.1 | Cathaya argyrophylla chloroplast, complete genome |
| NC_014570.1 | Eucalyptus grandis chloroplast, complete genome |
| NC_014697.1 | Prunus persica chloroplast, complete genome |
| NC_014699.1 | Equisetum arvense chloroplast, complete genome |
| NC_014674.1 | Castanea mollissima chloroplast, complete genome |
| NC_014675.1 | Isoetes flaccida chloroplast, complete genome |
| NC_014807.1 | Corynocarpus laevigata chloroplast, complete genome |
| NC_014874.1 | Rhizanthella gardneri plastid, complete genome |
| NC_015083.1 | Erodium carvifolium chloroplast, complete genome |
| NC_015113.1 | Anthriscus cerefolium chloroplast, complete genome |
| NC_015084.1 | Coccomyxa sp. C-169 plastid, complete genome |
| NC_015139.1 | Brassica rapa subsp. pekinensis chloroplast, complete genome |
| NC_011153.4 | Pinus contorta chloroplast, complete genome |
| NC_011154.4 | Pinus gerardiana chloroplast, complete genome |
| NC_011155.4 | Pinus krempfii chloroplast, complete genome |
| NC_011156.4 | Pinus lambertiana chloroplast, complete genome |
| NC_011158.4 | Pinus monophylla chloroplast, complete genome |
| NC_011159.4 | Pinus nelsonii chloroplast, complete genome |
| NC_015206.1 | Fragaria vesca subsp. vesca chloroplast, complete genome |
| NC_015204.1 | Gossypium thurberi chloroplast, complete genome |
| NC_015308.1 | Hevea brasiliensis chloroplast, complete genome |
| NC_015402.1 | Ptilidium pulcherrimum chloroplast, complete genome |
| NC_015401.1 | Olea europaea subsp. europaea plastid, complete genome |
| NC_015359.1 | Chlorella variabilis plastid, complete genome |
| NC_015605.1 | Nelumbo lutea chloroplast, complete genome |
| NC_015610.1 | Nelumbo nucifera chloroplast, complete genome |
| NC_015604.1 | Olea europaea subsp. cuspidata chloroplast, complete genome |
| NC_015608.1 | Olea woodiana subsp. woodiana chloroplast, complete genome |
| NC_015621.1 | Ageratina adenophora chloroplast, complete genome |
| NC_015623.1 | Olea europaea subsp. maroccana chloroplast, complete genome |
| NC_015543.1 | Jacobaea vulgaris chloroplast, complete genome |
| NC_015645.1 | Schizomeris leibleinii chloroplast, complete genome |
| NC_015803.1 | Indocalamus longiauritus plastid, complete genome |
| NC_015817.1 | Phyllostachys edulis chloroplast, complete genome |
| NC_015820.1 | Acidosasa purpurea chloroplast, complete genome |
| NC_015826.1 | Phyllostachys nigra var. henonis chloroplast, complete genome |
| NC_015830.1 | Bambusa emeiensis chloroplast, complete genome |
| NC_015831.1 | Ferrocalamus rimosivaginus plastid, complete genome |
| NC_014676.2 | Theobroma cacao chloroplast, complete genome |
| NC_015891.1 | Spirodela polyrhiza chloroplast, complete genome |
| NC_015892.1 | Magnolia kwangsiensis chloroplast, complete genome |
| NC_015894.1 | Wolffiella lingulata chloroplast, complete genome |
| NC_015899.1 | Wolffia australiana chloroplast, complete genome |
| NC_015990.1 | Panicum virgatum chloroplast, complete genome |
| NC_015983.1 | Cucumis melo subsp. melo chloroplast, complete genome |
| NC_015996.1 | Pyrus pyrifolia chloroplast, complete genome |
| NC_016064.1 | Pseudotsuga sinensis var. wilsoniana chloroplast, complete genome |
| NC_016068.1 | Nicotiana undulata chloroplast, complete genome |
| NC_016058.1 | Larix decidua chloroplast, complete genome |
| NC_016063.1 | Cephalotaxus wilsoniana chloroplast DNA, complete sequence |
| NC_016065.1 | Taiwania cryptomerioides chloroplast DNA, complete sequence |
| NC_016069.1 | Picea morrisonicola chloroplast, complete genome |
| NC_016430.1 | Eleutherococcus senticosus chloroplast, complete genome |
| NC_016468.1 | Boea hygrometrica chloroplast, complete genome |
| NC_016471.1 | Neottia nidus-avis plastid, complete genome |
| NC_013707.2 | Olea europaea chloroplast, complete genome |
| NC_016668.1 | Gossypium raimondii chloroplast, complete genome |
| NC_016670.1 | Gossypium darwinii chloroplast, complete genome |
| NC_016690.1 | Gossypium tomentosum chloroplast, complete genome |
| NC_016692.1 | Gossypium herbaceum subsp. africanum chloroplast, complete genome |
| NC_016711.1 | Gossypium mustelinum chloroplast, complete genome |
| NC_016712.1 | Gossypium arboreum chloroplast, complete genome |
| NC_016736.1 | Ricinus communis chloroplast, complete genome |
| NC_016677.1 | Leersia tisserantii plastid, complete genome |
| NC_016699.1 | Phyllostachys propinqua chloroplast, complete genome |
| NC_016718.1 | Rhynchoryza subulata plastid, complete genome |
| NC_016727.1 | Silene vulgaris chloroplast, complete genome |
| NC_016728.1 | Silene noctiflora chloroplast, complete genome |
| NC_016729.1 | Silene conica chloroplast, complete genome |
| NC_016730.1 | Silene latifolia chloroplast, complete genome |
| NC_016733.1 | Pedinomonas minor chloroplast, complete genome |
| NC_016753.1 | Colocasia esculenta chloroplast, complete genome |
| NC_016921.1 | Pentactina rupicola chloroplast, complete genome |
| NC_016927.1 | Oryza meridionalis chloroplast, complete genome |
| NC_016433.2 | Sesamum indicum chloroplast, complete genome |
| NC_016986.1 | Ginkgo biloba chloroplast, complete genome |
| NC_016732.1 | Dunaliella salina chloroplast, complete genome |
| NC_016734.1 | Brassica napus chloroplast, complete genome |
| NC_017006.1 | Mankyua chejuensis chloroplast, complete genome |
| NC_017602.1 | Elaeis guineensis chloroplast, complete genome |
| NC_017609.1 | Phalaenopsis equestris chloroplast, complete genome |
| NC_017835.1 | Oryza rufipogon chloroplast, complete genome |
| NC_017894.1 | Gossypium gossypioides chloroplast, complete genome |
| NC_016708.2 | Millettia pinnata chloroplast, complete genome |
| NC_018051.1 | Vigna unguiculata chloroplast, complete genome |
| NC_018109.1 | Gossypium incanum chloroplast, complete genome |
| NC_018110.1 | Gossypium somalense chloroplast, complete genome |
| NC_018111.1 | Gossypium capitis-viridis chloroplast, complete genome |
| NC_018112.1 | Gossypium areysianum chloroplast, complete genome |
| NC_018113.1 | Gossypium robinsonii chloroplast, complete genome |
| NC_018117.1 | Datura stramonium chloroplast, complete genome |
| NC_018114.1 | Erycina pusilla chloroplast, complete genome |
| NC_018357.1 | Magnolia denudata chloroplast, complete genome |
| NC_018541.1 | Elodea canadensis chloroplast, complete genome |
| NC_018552.1 | Capsicum annuum chloroplast, complete genome |
| NC_018565.1 | Pachycladon enysii chloroplast, complete genome |
| NC_018766.1 | Fragaria vesca subsp. bracteata chloroplast, complete genome |
| NC_018767.1 | Fragaria mandshurica plastid, complete genome |
| NC_018569.1 | Trebouxiophyceae sp. MX-AZ01 chloroplast, complete genome |
| NC_019649.1 | Festuca ovina plastid, complete genome |
| NC_019648.1 | Festuca altissima plastid, complete genome |
| NC_019650.1 | Festuca pratensis plastid, complete genome |
| NC_019651.1 | Lolium multiflorum plastid, complete genome |
| NC_019601.1 | Fragaria chiloensis chloroplast, complete genome |
| NC_019602.1 | Fragaria virginiana chloroplast, complete genome |
| NC_019616.1 | Vaccinium macrocarpon plastid, complete genome |
| NC_019628.1 | Pellia endiviifolia chloroplast, complete genome |
| NC_020019.1 | Camellia sinensis chloroplast, complete genome |
| NC_020092.1 | Chrysanthemum x morifolium chloroplast, complete genome |
| NC_020098.1 | Tectona grandis chloroplast, complete genome |
| NC_020147.1 | Ophioglossum californicum chloroplast, complete genome |
| NC_020152.1 | Quercus rubra plastid, complete genome |
| NC_020146.1 | Equisetum hyemale chloroplast, complete genome |
| NC_020259.1 | Nothoceros aenigmaticus plastid sequence |
| NC_020316.1 | Magnolia officinalis chloroplast, complete genome |
| NC_020317.1 | Magnolia officinalis subsp. biloba voucher SC001 chloroplast, complete genome |
| NC_020318.1 | Magnolia grandiflora voucher NJ016 chloroplast, complete genome |
| NC_020319.1 | Cycas revoluta chloroplast, complete genome |
| NC_020320.1 | Chrysanthemum indicum voucher HeN001 chloroplast, complete genome |
| NC_020321.1 | Taxus mairei voucher NN014 chloroplast, complete genome |
| NC_020341.1 | Arundinaria gigantea chloroplast, complete genome |
| NC_020361.1 | Podocarpus totara chloroplast, complete genome |
| NC_020362.1 | Heliconia collinsiana plastid, complete genome |
| NC_020363.1 | Zingiber spectabile plastid, complete genome |
| NC_020364.1 | Pseudophoenix vinifera plastid, complete genome |
| NC_020366.1 | Bismarckia nobilis plastid, complete genome |
| NC_020367.1 | Dasypogon bromeliifolius plastid, complete genome |
| NC_020365.1 | Calamus caryotoides plastid, complete genome |
| NC_020372.1 | Trithuria inconspicua complete chloroplast genome |
| NC_020431.1 | Salvia miltiorrhiza chloroplast, complete genome |
| NC_020438.1 | Gonium pectorale chloroplast, complete genome |
| NC_020607.1 | Artemisia frigida chloroplast, complete genome |
| NC_021121.1 | Ardisia polysticta chloroplast, complete genome |
| NC_021102.1 | Pachycladon cheesemanii chloroplast, complete genome |
| NC_021109.1 | Pleodorina starrii plastid, complete genome |
| NC_021091.1 | Vigna angularis chloroplast DNA, complete sequence |
| NC_021110.1 | Cephalotaxus oliveri chloroplast, complete genome |
| NC_021111.1 | Cistanche deserticola chloroplast, complete genome |
| NC_021101.1 | Francoa sonchifolia plastid, complete genome |
| NC_021372.1 | Pharus latifolius chloroplast, complete genome |
| NC_021423.1 | Catharanthus roseus cultivar Pacifica Punch Halo chloroplast, complete genome |
| NC_021425.1 | Tetracentron sinense chloroplast, complete genome |
| NC_021426.1 | Trochodendron aralioides chloroplast, complete genome |
| NC_021430.1 | Cymbidium sinense voucher KUN:YJB100605 chloroplast, complete genome |
| NC_021432.1 | Cymbidium tracyanum voucher KUN:YJB100606 chloroplast, complete genome |
| NC_021433.1 | Cymbidium mannii voucher KUN:YJB100602 chloroplast, complete genome |
| NC_021455.1 | Prinsepia utilis plastid, complete genome |
| NC_021431.1 | Cymbidium tortisepalum voucher KUN:HJL091027 chloroplast, complete genome |
| NC_021437.1 | Cunninghamia lanceolata chloroplast, complete genome |
| NC_021438.1 | Gnetum montanum chloroplast, complete genome |
| NC_021439.1 | Pinus massoniana chloroplast, complete genome |
| NC_021440.1 | Pinus taeda chloroplast, complete genome |
| NC_021441.1 | Taiwania flousiana chloroplast, complete genome |
| NC_021449.1 | Utricularia gibba chloroplast, complete genome |
| NC_021429.1 | Cymbidium aloifolium voucher KUN:YJB100604 chloroplast, complete genome |
| NC_021636.1 | Glycine tomentella voucher CSIRO:G1403 chloroplast, complete genome |
| NC_021456.1 | Picea abies chloroplast complete genome |
| NC_021760.1 | Triticum monococcum chloroplast, complete genome |
| NC_021761.1 | Secale cereale chloroplast, complete genome |
| NC_021762.1 | Triticum urartu chloroplast, complete genome |
| NC_021645.1 | Glycine cyrtoloba voucher CSIRO:G1267 chloroplast, complete genome |
| NC_021646.1 | Glycine stenophita voucher CSIRO:G1974 chloroplast, complete genome |
| NC_021647.1 | Glycine canescens voucher CSIRO:G1232 chloroplast, complete genome |
| NC_021648.1 | Glycine dolichocarpa voucher CSIRO:G1134 chloroplast, complete genome |
| NC_021649.1 | Glycine falcata voucher CSIRO:G1718 chloroplast, complete genome |
| NC_021650.1 | Glycine syndetika voucher CSIRO:G1300 chloroplast, complete genome |
| NC_021936.1 | Najas flexilis chloroplast, complete genome |
| NC_022133.1 | Aegilops tauschii chloroplast, complete genome |
| NC_022135.1 | Aegilops speltoides isolate SPE0661 chloroplast, complete genome |
| NC_022136.1 | Lygodium japonicum chloroplast, complete genome |
| NC_022137.1 | Marsilea crenata chloroplast, complete genome |
| NC_022264.1 | Camellia taliensis voucher HKAS:S.X.Yang3157 chloroplast, complete genome |
| NC_007898.3 | Solanum lycopersicum chloroplast, complete genome |
| NC_022378.1 | Eucalyptus obliqua chloroplast, complete genome |
| NC_022379.1 | Eucalyptus radiata chloroplast, complete genome |
| NC_022380.1 | Eucalyptus delegatensis chloroplast, complete genome |
| NC_022381.1 | Eucalyptus verrucata chloroplast, complete genome |
| NC_022382.1 | Eucalyptus baxteri chloroplast, complete genome |
| NC_022383.1 | Eucalyptus diversifolia chloroplast, complete genome |
| NC_022384.1 | Eucalyptus sieberi chloroplast, complete genome |
| NC_022385.1 | Eucalyptus elata chloroplast, complete genome |
| NC_022386.1 | Eucalyptus regnans chloroplast, complete genome |
| NC_022387.1 | Eucalyptus umbra chloroplast, complete genome |
| NC_022388.1 | Eucalyptus cloeziana chloroplast, complete genome |
| NC_022389.1 | Eucalyptus patens chloroplast, complete genome |
| NC_022390.1 | Eucalyptus marginata chloroplast, complete genome |
| NC_022391.1 | Eucalyptus curtisii chloroplast, complete genome |
| NC_022392.1 | Eucalyptus melliodora chloroplast, complete genome |
| NC_022393.1 | Eucalyptus polybractea chloroplast, complete genome |
| NC_022394.1 | Eucalyptus cladocalyx chloroplast, complete genome |
| NC_022395.1 | Eucalyptus nitens chloroplast, complete genome |
| NC_022396.1 | Eucalyptus aromaphloia chloroplast, complete genome |
| NC_022397.1 | Eucalyptus saligna chloroplast, complete genome |
| NC_022398.1 | Eucalyptus camaldulensis chloroplast, complete genome |
| NC_022399.1 | Eucalyptus deglupta chloroplast, complete genome |
| NC_022400.1 | Eucalyptus spathulata chloroplast, complete genome |
| NC_022401.1 | Eucalyptus torquata chloroplast, complete genome |
| NC_022402.1 | Eucalyptus diversicolor chloroplast, complete genome |
| NC_022403.1 | Eucalyptus salmonophloia chloroplast, complete genome |
| NC_022404.1 | Eucalyptus microcorys chloroplast, complete genome |
| NC_022405.1 | Eucalyptus guilfoylei chloroplast, complete genome |
| NC_022406.1 | Eucalyptus erythrocorys chloroplast, complete genome |
| NC_022407.1 | Corymbia gummifera chloroplast, complete genome |
| NC_022408.1 | Corymbia maculata chloroplast, complete genome |
| NC_022409.1 | Corymbia eximia chloroplast, complete genome |
| NC_022410.1 | Corymbia tessellaris chloroplast, complete genome |
| NC_022411.1 | Angophora floribunda chloroplast, complete genome |
| NC_022412.1 | Angophora costata chloroplast, complete genome |
| NC_022414.1 | Stockwellia quadrifida chloroplast, complete genome |
| NC_022431.1 | Asclepias nivea chloroplast, complete genome |
| NC_022413.1 | Allosyncarpia ternata chloroplast, complete genome |
| NC_022432.1 | Asclepias syriaca chloroplast, complete genome |
| NC_022417.1 | Cocos nucifera chloroplast, complete genome |
| NC_022459.1 | Camellia cuspidata voucher HKAS:S.X.Yang3159 chloroplast, complete genome |
| NC_022460.1 | Camellia danzaiensis voucher HKAS:S.X.Yang3147 chloroplast, complete genome |
| NC_022461.1 | Camellia impressinervis voucher HKAS:S.X.Yang1080 chloroplast, complete genome |
| NC_022463.1 | Camellia yunnanensis voucher HKAS:S.X.Yang1090 chloroplast, complete genome |
| NC_022451.1 | Andrographis paniculata chloroplast, complete genome |
| NC_022457.1 | Berberis bealei chloroplast, complete genome |
| NC_022462.1 | Camellia pitardii voucher HKAS:S.X.Yang3148 chloroplast, complete genome |
| NC_022668.1 | Oryza rufipogon chloroplast, complete genome |
| NC_022811.1 | Brassaiopsis hainla chloroplast, complete genome |
| NC_022812.1 | Metapanax delavayi chloroplast, complete genome |
| NC_022813.1 | Schefflera delavayi chloroplast, complete genome |
| NC_022850.1 | Setaria italica plastid, complete genome |
| NC_022859.1 | Lindenbergia philippensis chloroplast complete genome |
| NC_022868.1 | Glycine soja chloroplast, complete genome |
| NC_022958.1 | Phragmites australis chloroplast, complete genome |
| NC_022810.1 | Aralia undulata chloroplast, complete genome |
| NC_022814.1 | Kalopanax septemlobus chloroplast, complete genome |
| NC_022926.1 | Musa textilis plastid, complete genome |
| NC_022927.1 | Ravenala madagascariensis plastid, complete genome |
| NC_022928.1 | Curcuma roscoeana plastid, complete genome |
| NC_023084.1 | Camellia oleifera plastid, complete genome |
| NC_023085.1 | Sedum sarmentosum chloroplast, complete genome |
| NC_023086.1 | Penthorum chinense chloroplast, complete genome |
| NC_023090.1 | Lupinus luteus chloroplast, complete genome |
| NC_023092.1 | Liquidambar formosana chloroplast, complete genome |
| NC_023097.1 | Aegilops geniculata chloroplast, complete genome |
| NC_023109.1 | Helianthus divaricatus isolate DB07 plastid, complete genome |
| NC_023110.1 | Helianthus decapetalus isolate DB11 plastid, complete genome |
| NC_023113.1 | Helianthus strumosus isolate DB31 plastid, complete genome |
| NC_023114.1 | Helianthus maximiliani isolate MAX01 plastid, complete genome |
| NC_023119.1 | Agathis dammara chloroplast DNA, complete genome |
| NC_023121.1 | Calocedrus formosana chloroplast DNA, complete genome |
| NC_023130.1 | Pyrus spinosa chloroplast, isolate PYR002, complete sequence |
| NC_023131.1 | Conopholis americana chloroplast complete genome |
| NC_023132.1 | Phelipanche purpurea chloroplast complete genome, specimen voucher BONN:S. Wicke Op38/39 |
| NC_023096.1 | Aegilops cylindrica chloroplast, complete genome |
| NC_023102.1 | Ajuga reptans plastid, complete genome |
| NC_023107.1 | Helianthus giganteus isolate DB01 plastid, complete genome |
| NC_023108.1 | Helianthus grosseserratus isolate DB05 plastid, complete genome |
| NC_023111.1 | Helianthus hirsutus isolate DB14 plastid, complete genome |
| NC_023112.1 | Helianthus tuberosus isolate DB16 plastid, complete genome |
| NC_023120.1 | Nageia nagi chloroplast DNA, complete genome |
| NC_023245.1 | Pharus lappulaceus chloroplast, complete genome |
| NC_023247.1 | Fritillaria taipaiensis chloroplast, complete genome |
| NC_023213.1 | Gossypium anomalum chloroplast, complete genome |
| NC_023214.1 | Gossypium bickii chloroplast, complete genome |
| NC_023218.1 | Gossypium sturtianum chloroplast, complete genome |
| NC_023215.1 | Gossypium herbaceum chloroplast, complete genome |
| NC_023216.1 | Gossypium longicalyx chloroplast, complete genome |
| NC_023234.1 | Magnolia cathcartii chloroplast, complete genome |
| NC_023235.1 | Magnolia dealbata chloroplast, complete genome |
| NC_023236.1 | Magnolia pyramidata chloroplast, complete genome |
| NC_023237.1 | Magnolia kobus chloroplast, complete genome |
| NC_023238.1 | Magnolia liliifera chloroplast, complete genome |
| NC_023239.1 | Michelia odora chloroplast, complete genome |
| NC_023241.1 | Magnolia sinica chloroplast, complete genome |
| NC_023242.1 | Magnolia sprengeri chloroplast, complete genome |
| NC_023217.1 | Gossypium stocksii chloroplast, complete genome |
| NC_023256.1 | Melianthus villosus plastid, complete genome |
| NC_023259.1 | Viviania marifolia chloroplast, complete genome |
| NC_023260.1 | Hypseocharis bilobata chloroplast, complete genome |
| NC_023261.1 | Pelargonium alternans chloroplast, complete genome |
| NC_023240.1 | Magnolia salicifolia chloroplast, complete genome |
| NC_023115.1 | Schwalbea americana chloroplast genome |
| NC_023367.1 | Arabis alpina complete chloroplast genome |
| NC_023356.1 | Petrosavia stellaris plastid, complete genome |
| NC_023358.1 | Silene conoidea chloroplast, complete genome |
| NC_023357.1 | Agrostemma githago chloroplast, complete genome |
| NC_023360.1 | Silene paradoxa chloroplast, complete genome |
| NC_023359.1 | Silene chalcedonica chloroplast, complete genome |
| NC_022715.2 | Veratrum patulum chloroplast, complete genome |
| NC_023465.1 | Phelipanche ramosa chloroplast complete genome |
| NC_023449.1 | Puelia olyriformis chloroplast, complete genome |
| NC_023463.1 | Pinguicula ehlersiae chloroplast, complete genome |
| NC_023464.1 | Orobanche gracilis chloroplast, complete genome |
| NC_023533.1 | Deschampsia antarctica plastid, complete genome |
| NC_023544.1 | Cucumis hystrix plastid, complete genome |
| NC_023775.1 | Auxenochlorella protothecoides chloroplast, complete genome |
| NC_023790.1 | Vitis rotundifolia chloroplast, complete genome |
| NC_023792.1 | Azadirachta indica chloroplast, complete genome |
| NC_023798.1 | Prunus mume isolate BJFU1210120008 plastid, complete genome |
| NC_023800.1 | Sorghum timorense chloroplast, complete genome |
| NC_023801.1 | Castanopsis echinocarpa chloroplast, complete genome |
| NC_023805.1 | Podocarpus lambertii chloroplast, complete genome |
| NC_023833.1 | Praxelis clematidea chloroplast, complete genome |
| NC_023835.1 | Chlorella sorokiniana chloroplast, complete genome |
| NC_023934.1 | Arundinaria appalachiana chloroplast, complete genome |
| NC_023935.1 | Arundinaria tecta chloroplast, complete genome |
| NC_023959.1 | Trigonobalanus doichangensis chloroplast, complete genome |
| NC_024019.1 | Dendrobium officinale chloroplast, complete genome |
| NC_024022.1 | Juniperus monosperma chloroplast, complete genome |
| NC_024023.1 | Juniperus scopulorum chloroplast, complete genome |
| NC_024024.1 | Juniperus virginiana chloroplast, complete genome |
| NC_024027.1 | Magnolia tripetala plastid, complete genome |
| NC_024021.1 | Juniperus bermudiana chloroplast, complete genome |
| NC_024035.1 | Trifolium aureum plastid, complete genome |
| NC_024036.1 | Trifolium repens plastid, complete genome |
| NC_023956.1 | Prunus kansuensis chloroplast, complete genome |
| NC_024034.1 | Trifolium grandiflorum plastid, complete genome |
| NC_024038.1 | Glycyrrhiza glabra chloroplast, complete genome |
| NC_024060.1 | Hirtella racemosa chloroplast, complete genome |
| NC_024061.1 | Chrysobalanus icaco chloroplast, complete genome |
| NC_024062.1 | Licania heteromorpha chloroplast, complete genome |
| NC_024064.1 | Licania alba chloroplast, complete genome |
| NC_024065.1 | Licania sprucei chloroplast, complete genome |
