# Supplementary material for: Complete chloroplast genome sequence of MD-2 pineapple and its comparative analysis among nine other plants from the subclass Commelinidae
Source: BMC Plant Biol. 2015 Aug 12;15:196. doi: 10.1186/s12870-015-0587-1 (PMC4534033; doi:10.1186/s12870-015-0587-1)
Supplement: Additional file 4: — Complete list of the SSRs identified in the chloroplast of pineapple. SSRs identified using Misa in A. comosus chloroplast genome. (DOCX 38 kb) [file 12870_2015_587_MOESM4_ESM.docx]

Table 1: SSR identified in the chloroplast of pineapple.

| No SSR | SSR Type | SSR | Size | Start | End | Location |
| --- | --- | --- | --- | --- | --- | --- |
| 1 | p1 | (A)8 | 8 | 2912 | 2919 | LSC (*matK*) |
| 2 | p1 | (A)9 | 9 | 3552 | 3560 | LSC |
| 3 | p4 | (TTTA)3 | 12 | 4723 | 4734 | LSC |
| 4 | p2 | (TA)4 | 8 | 5544 | 5551 | LSC (*rps16*) |
| 5 | p1 | (A)9 | 9 | 6312 | 6320 | LSC |
| 6 | p2 | (AT)4 | 8 | 6580 | 6587 | LSC |
| 7 | p1 | (T)9 | 9 | 6787 | 6795 | LSC |
| 8 | p1 | (A)12 | 12 | 8296 | 8307 | LSC (*psbI*) |
| 9 | c | (TTTA)3tttttatttaatcttatttaactttaatattattattta(ATT)4 | 63 | 8748 | 8810 | LSC |
| 10 | p2 | (AT)8 | 16 | 9002 | 9017 | LSC |
| 11 | c | (CAA)4gagaaatttccattatcattcctatgatcgaagtcccatttattattatttaatttaatatttcttttttattcttc(T)9attgaagattctttttttctttttctcagtttattacttaatttcttactgttgtcaagtaaggaataaaaaaacacatatgataacatatc(AT)5 | 200 | 9317 | 9516 | LSC |
| 12 | p1 | (T)10 | 10 | 10107 | 10116 | LSC |
| 13 | p2 | (TC)4 | 8 | 10546 | 10553 | LSC |
| 14 | p1 | (T)8 | 8 | 11004 | 11011 | LSC |
| 15 | p1 | (T)10 | 10 | 13265 | *13274* | LSC (**atpH*) |
| 16 | p1 | (A)8 | 8 | 13983 | 13990 | LSC (*atpH*) |
| 17 | p4 | (ATTT)3 | 12 | 14196 | 14207 | LSC |
| 18 | p1 | (A)8 | 8 | 14821 | 14828 | LSC |
| 19 | p4 | (TTTC)3 | 12 | 15038 | 15049 | LSC |
| 20 | c* | (AT)6tttttattttgctataactaac(TA)6ttatatatgtatagcatatagttag(AT)4(TAGT)3*atataggtagggtataaggtagggtataaggacttatatt(TA)4 | 138 | 15305 | 15442 | LSC |
| 21 | p1 | (T)12 | 12 | 15631 | 15642 | LSC |
| 22 | p1 | (A)9 | 9 | 15768 | 15776 | LSC |
| 23 | c | (AT)4caatatccccagttc(T)10 | 33 | 16665 | 16697 | LSC |
| 24 | c | (T)10ccgtttctggtggtataaaaatgccgctgtgcctggatatcttatctgtctctccaggaaaatgaatatctccagaaaagattttaagttcaatata(T)10 | 117 | 19655 | 19771 | LSC |
| 25 | p1 | (A)8 | 8 | 19910 | 19917 | LSC |
| 26 | c | (AT)4tggttgtgctcgaaaggttatgaatcgattgacaagtccaatcccaatatcttgatttcgagtggcaatgcatcgtagaccg(AT)5 | 100 | 21044 | 21143 | LSC |
| 27 | p1 | (A)8 | 8 | 23582 | 23589 | LSC (*rpoC1*) |
| 28 | p1 | (T)10 | 10 | 23875 | 23884 | LSC (**rpoC1*) |
| 29 | p1 | (T)8 | 8 | 27428 | 27435 | LSC (*rpoB*) |
| 30 | p1 | (A)14 | 14 | 28556 | 28569 | LSC |
| 31 | c | (AG)4acccgactcggtatctgtgtaccaatttctgttctggggtttacatatac(AT)4 | 66 | 28830 | 28895 | LSC |
| 32 | p1 | (A)10 | 10 | 29091 | 29100 | LSC |
| 33 | p2 | (GT)4 | 8 | 29229 | 29236 | LSC |
| 34 | c | (A)8gatttctttggtcgtgtagggagattac(A)8 | 44 | 30092 | 30135 | LSC |
| 35 | p2 | (GT)4 | 8 | 30364 | 30371 | LSC |
| 36 | c | (A)11ttccttggttttgttttcttttgctatagtatattcccatactattcttcctctattgattctttcgatggatcccggaacc(TA)4 | 101 | 31346 | 31446 | LSC |
| 37 | p2 | (TA)4 | 8 | 31744 | 31751 | LSC |
| 38 | p2 | (CA)4 | 8 | 32362 | 32369 | LSC |
| 39 | p2 | (AG)4 | 8 | 32990 | 32997 | LSC |
| 40 | p1 | (A)11 | 11 | 33424 | 33434 | LSC |
| 41 | p1 | (T)11 | 11 | 33866 | 33876 | LSC |
| 42 | c | (A)10tggctaattcatgaattgaataaaacggcccttttaactcagcggtagagtaacgccatggtaaggcgtaagtcatcggttcaaatccgataaagggc(T)9 | 117 | 34021 | 34137 | LSC |
| 43 | p1 | (A)13 | 13 | 35034 | 35046 | LSC |
| 44 | p1 | (G)8 | 8 | 36493 | 36500 | LSC(*psbC*) |
| 45 | p1 | (G)8 | 8 | 36764 | 36771 | LSC(*psbC*) |
| 46 | p2 | (GA)4 | 8 | 37752 | 37759 | LSC |
| 47 | p1 | (A)9 | 9 | 38455 | 38463 | LSC |
| 48 | p1 | (T)10 | 10 | 44673 | 44682 | LSC |
| 49 | p1 | (T)10 | 10 | 45435 | 45444 | LSC(**ycf3*) |
| 50 | p1 | (A)8 | 8 | 46536 | 46543 | LSC(**ycf3*) |
| 51 | p1 | (A)14 | 14 | 47541 | 47554 | LSC |
| 52 | c | (T)10attttattgaaatttcattttacacaataaatctaaattcaaactgaactaaaggataaacaaagcaaaatcgactgatgaagtactac(A)8tgaattgtatcaacatctggattttttg(TA)6 | 147 | 48292 | 48438 | LSC |
| 53 | p1 | (A)11 | 11 | 48832 | 48842 | LSC |
| 54 | p1 | (A)13 | 13 | 49164 | 49176 | LSC |
| 55 | p2 | (AG)4 | 8 | 50027 | 50034 | LSC |
| 56 | p2 | (AT)4 | 8 | 50763 | 50770 | LSC |
| 57 | p1 | (T)8 | 8 | 50894 | 50901 | LSC |
| 58 | p1 | (T)11 | 11 | 52531 | 52541 | LSC (*ndhK*) |
| 59 | p1 | (T)12 | 12 | 53770 | 53781 | LSC |
| 60 | c | (T)12c(A)9 | 22 | 57115 | 57136 | LSC |
| 61 | p2 | (TA)4 | 8 | 57493 | 57500 | LSC |
| 62 | c | (A)8ggattgagccgaataaaataaagaatgagcttactaacataacatactatatatttgcatatatctttcatatcacatgtacagacctatatac(TA)4 | 110 | 59242 | 59351 | LSC |
| 63 | c | (A)12gaatctatctatatagatagatattgaagtgatatctcggattcc(A)8 | 65 | 59679 | 59743 | LSC |
| 64 | p1 | (T)8 | 8 | 60235 | 60242 | LSC (*accD*) |
| 65 | p2 | (TG)4 | 8 | 60967 | 60974 | LSC (*accD*) |
| 66 | c | (A)8ctaagaaaaatgttctttggtgatataagttacactttctagtaagagtcagaagtttcggataa(T)8 | 81 | 61424 | 61504 | LSC |
| 67 | p2 | (TA)4 | 8 | 61756 | 61763 | LSC |
| 68 | p1 | (T)11 | 11 | 62023 | 62033 | LSC |
| 69 | c | (TA)4cttaattgtt(TA)4 | 26 | 62150 | 62175 | LSC |
| 70 | c | (CA)4aatgaaagaactgttatgcatgcggatacatgatatccgcataaatgcatg(TA)4 | 67 | 62521 | 62587 | LSC |
| 71 | p1 | (T)8 | 8 | 62943 | 62950 | LSC (*ycf4*) |
| 72 | c | (A)8gaaagcattgccttctttcccatatcttgtatctatagtatttttgccctggtggg(TC)5 | 74 | 64148 | 64221 | LSC (*cemA*) |
| 73 | p4 | (AATG)3 | 12 | 64822 | 64833 | LSC (*cemA*) |
| 74 | c | (C)8gatcgtatttctcctgagttgaaagaaaagataggaaatctgtcttttcagagttatcgtcccaat(A)8 | 82 | 65420 | 65501 | LSC (*petA*) |
| 75 | p1 | (T)11 | 11 | 66366 | 66376 | LSC (*petA*) |
| 76 | c | (A)11taattaataactataattagtaactat(TAA)4ataaaa(AAT)4aaataaaaaaagaatatataatatat(A)9g(AT)11aatagtagataatatataaattagtagataatatataa(AT)4aatagtagataatagtagtagataatagtaatagtatctactatttagattagatcatatttaat(GAAA)3 | 249 | 68107 | 68355 | LSC |
| 77 | p1 | (T)9 | 9 | 68947 | 68955 | LSC |
| 78 | p1 | (A)14 | 14 | 69118 | 69131 | LSC |
| 79 | p1 | (T)8 | 8 | 69396 | 69403 | LSC |
| 80 | p1 | (T)16 | 16 | 69748 | 69763 | LSC |
| 81 | p1 | (A)8 | 8 | 70604 | 70611 | LSC |
| 82 | c* | (T)8(TTC)4* | 18 | 70886 | 70903 | LSC |
| 83 | p4 | (AATA)3 | 12 | 71356 | 71367 | LSC |
| 84 | p1 | (T)9 | 9 | 71835 | 71843 | LSC |
| 85 | c | (TA)4tg(TA)4 | 18 | 73152 | 73169 | LSC |
| 86 | p1 | (A)9 | 9 | 73620 | 73628 | LSC (**clpP*) |
| 87 | p1 | (T)11 | 11 | 73898 | 73908 | LSC (**clpP*) |
| 88 | p1 | (T)10 | 10 | 74168 | 74177 | LSC (**clpP*) |
| 89 | c | (A)8ttgcg(A)11gaatcaatgtgtcgattccagttttatttc(T)10atgtaacaggtttttttaatgaaaggtcttctatt(A)8cgagatgggttttggctcccttccctct(A)9 | 144 | 74576 | 74719 | LSC (**clpP*) |
| 90 | p1 | (T)11 | 11 | 74920 | 74930 | LSC (**clpP*) |
| 91 | p4 | (TGAT)3 | 12 | 75256 | 75267 | LSC (**clpP*) |
| 92 | p2 | (TA)4 | 8 | 75816 | 75823 | LSC |
| 93 | p1 | (T)8 | 8 | 76623 | 76630 | LSC (*psbB*) |
| 94 | p1 | (T)10 | 10 | 77448 | 77457 | LSC |
| 95 | p1 | (A)8 | 8 | 78546 | 78553 | LSC |
| 96 | p1 | (A)10 | 10 | 78824 | 78833 | LSC |
| 97 | p1 | (T)13 | 13 | 83242 | 83254 | LSC |
| 98 | c | (T)11c(T)11 | 23 | 84099 | 84121 | LSC |
| 99 | p1 | (A)13 | 13 | 84619 | 84631 | LSC |
| 100 | c | (GT)4acatttatgtatctttatagataatgtaatgtttctcttttttatttttataacataacgaatcctttcctta(T)8 | 89 | 85327 | 85415 | LSC |
| 101 | c | (TTCT)3atcatccttccatttatccatatcccttta(T)12 | 54 | 85876 | 85929 | LSC |
| 102 | p1 | (T)14 | 14 | 86108 | 86121 | LSC |
| 103 | p1 | (T)13 | 13 | 86235 | 86247 | LSC |
| 104 | p1 | (T)9 | 9 | 86770 | 86778 | LSC (*rpl22*) |
| 105 | c | (TA)6(T)13 | 25 | 86985 | 87009 | LSC |
| 106 | c | (T)9agtgaacgtgtcacagctgattactcc(T)9 | 45 | 87777 | 87821 | IRA (*rps19*) |
| 107 | c | (GA)4tatt(GA)4 | 20 | 90192 | 90211 | IRA (*ycf2)* |
| 108 | p2 | (GA)4 | 8 | 91179 | 91186 | IRA (*ycf2)* |
| 109 | p1 | (A)8 | 8 | 92081 | 92088 | IRA (*ycf2)* |
| 110 | c | (A)9catatttttttg(GA)5 | 31 | 93364 | 93394 | IRA (*ycf2)* |
| 111 | p2 | (TA)4 | 8 | 98303 | 98310 | IRA |
| 112 | p2 | (AG)4 | 8 | 99049 | 99056 | IRA (*ndhB*) |
| 113 | p1 | (T)9 | 9 | 102993 | 103001 | IRA |
| 114 | p1 | (T)8 | 8 | 106868 | 106875 | IRA (*ycf68*) |
| 115 | p2 | (CT)4 | 8 | 110190 | 110197 | IRA |
| 116 | p1 | (A)8 | 8 | 114267 | 114274 | SSC (*ycf1)* |
| 117 | c | (A)9gatat(A)13 | 27 | 116577 | 116603 | SSC |
| 118 | p1 | (T)9 | 9 | 116827 | 116835 | SSC |
| 119 | p1 | (A)14 | 14 | 116980 | 116993 | SSC |
| 120 | c | (T)9cccctcatgttgtcatttatatatgtgatgtgtgat(GC)4atacatatattg(AT)5cacatatacatg(TA)4aatatatttgtat(TA)4 | 116 | 117293 | 117408 | SSC |
| 121 | p1 | (T)11 | 11 | 117918 | 117928 | SSC |
| 122 | p4 | (AATA)3 | 12 | 120114 | 120125 | SSC (*ndhD*) |
| 123 | p4 | (TATT)3 | 12 | 122614 | 122625 | SSC |
| 124 | p1 | (T)12 | 12 | 123314 | 123325 | SSC |
| 125 | p1 | (A)9 | 9 | 123443 | 123451 | SSC |
| 126 | p1 | (A)8 | 8 | 124880 | 124887 | SSC (**ndhA)* |
| 127 | p1 | (A)10 | 10 | 124991 | 125000 | SSC (**ndhA)* |
| 128 | c | (T)12attattcattcaactactgcattccatttcttttttcctgttcttctgtctcagaggaggatact(A)10 | 87 | 125187 | 125273 | SSC (**ndhA)* |
| 129 | p1 | (A)17 | 17 | 125754 | 125770 | SSC (**ndhA)* |
| 130 | p1 | (A)8 | 8 | 127669 | 127676 | SSC |
| 131 | p1 | (A)11 | 11 | 128022 | 128032 | SSC |
| 132 | p2 | (AT)4 | 8 | 128328 | 128335 | SSC |
| 133 | p1 | (T)8 | 8 | 128775 | 128782 | SSC (*ycf1*) |
| 134 | c | (T)9gttttcttttcgtatatctgatacaaaatttacttggtcctgttgttcatttttttgttggttggaattttctaatttaagata(T)8 | 101 | 129205 | 129305 | SSC (*ycf1*) |
| 135 | c | (A)8tacttttttgattggatgggttgatttcttgatgaatcgtaagagaaaaaatatc(T)9 | 72 | 129526 | 129597 | SSC (*ycf1*) |
| 136 | p1 | (T)8 | 8 | 130524 | 130531 | SSC (*ycf1*) |
| 137 | p1 | (T)8 | 8 | 131068 | 131075 | SSC (*ycf1*) |
| 138 | p1 | (T)10 | 10 | 131188 | 131197 | SSC (*ycf1*) |
| 139 | p1 | (T)8 | 8 | 131570 | 131577 | SSC (*ycf1*) |
| 140 | p1 | (A)10 | 10 | 131694 | 131703 | SSC (*ycf1*) |
| 141 | p1 | (A)8 | 8 | 132780 | 132787 | SSC (*ycf1*) |
| 142 | p2 | (AG)4 | 8 | 136922 | 136929 | IRB |
| 143 | p1 | (A)8 | 8 | 140244 | 140251 | IRB(*ycf68*) |
| 144 | p1 | (A)9 | 9 | 144118 | 144126 | IRB |
| 145 | p2 | (CT)4 | 8 | 148063 | 148070 | IRB (*ndhB*) |
| 146 | p2 | (AT)4 | 8 | 148808 | 148815 | IRB |
| 147 | c | (TC)5caaaaaaatatg(T)9 | 31 | 153725 | 153755 | IRB |
| 148 | p1 | (T)8 | 8 | 155031 | 155038 | IRB |
| 149 | p2 | (TC)4 | 8 | 155933 | 155940 | IRB (*ycf2*) |
| 150 | c | (TC)4aata(TC)4 | 20 | 156908 | 156927 | IRB (*ycf2*) |
| 151 | c | (A)9ggagtaatcagctgtgacacgttcact(A)9 | 45 | 159298 | 159342 | IRB (*rps19*) |
| ­ |  |  |  |  |  |  |
| Gene name in parenthesis indicates SSR that is coded within the CDS of the respective gene | | | | | | |
| * SSR in the intronic region of the respective gene in parenthesis | | |  |  |  |  |
