# Supplementary material for: Complete chloroplast genome sequence of MD-2 pineapple and its comparative analysis among nine other plants from the subclass Commelinidae
Source: BMC Plant Biol. 2015 Aug 12;15:196. doi: 10.1186/s12870-015-0587-1 (PMC4534033; doi:10.1186/s12870-015-0587-1)
Supplement: Additional file 5: — Estimates of average evolutionary divergence over 80 protein coding-gene sequences from the subclass commelinid. Standard error estimate(s) are shown in the second column and were obtained by a bootstrap procedure (1000 replicates). Analyses were conducted using the Kimura 2-parameter model [1]. The analysis involved 100 nucleotide sequences. Codon positions included were 1st + 2nd + 3rd + Noncoding. All positions with less than 95 % site coverage were eliminated. That is, fewer than 5 % alignment gaps, missing data, and ambiguous bases were allowed at any position. There were a total of 465 positions in the final dataset. Evolutionary analyses were conducted in MEGA6. (DOCX 29 kb) [file 12870_2015_587_MOESM5_ESM.docx]

Table 1:

| **Gene** | **Location** | **d** | **S.E** | **Average** | **Range** | **Taxa with respective gene lost/number of taxa with respective gene lost** | **Functional Group** |
| --- | --- | --- | --- | --- | --- | --- | --- |
| ndhB | IR | 0.009 | 0.001 | 1535.04 | 1526-1550 | 0 | NADH-dehydrogenase |
| psbL | LSC | 0.012 | 0.003 | 119.6 | 117-182 | 0 | Photosystem II |
| rps7 | IR | 0.02 | 0.003 | 473.72 | 468-590 | 0 | Small ribosomal subunit |
| psbE | LSC | 0.021 | 0.005 | 253.98 | 252-254 | 0 | Photosystem II |
| atpH | LSC | 0.026 | 0.004 | 247.98 | 246-248 | 0 | ATP synthase |
| petG | LSC | 0.028 | 0.008 | 115.98 | 114-116 | 0 | Cytochrome b/f complex |
| petN | LSC | 0.029 | 0.008 | 91.98 | 90-92 | 0 | Cytochrome b/f complex |
| rpl2 | IR | 0.032 | 0.003 | 788.53 | 317-839 | *Aegilops cylindrica.* | Large ribosomal subunit |
| ycf3 | LSC | 0.032 | 0.004 | 518.63 | 509-521 | 0 | Photosystem I |
| psaB | LSC | 0.034 | 0.002 | 2207.12 | 2205-2219 | *Lecomtella madagascariensis* | Photosystem I |
| psaJ | LSC | 0.035 | 0.008 | 132.22 | 128-137 | *Brachypodium distachyon Oryza glaberrima Oryza sativa Japonica* | Photosystem I |
| psbT | LSC | 0.035 | 0.01 | 111.17 | 104-119 | 0 | Photosystem II |
| petL | LSC | 0.036 | 0.008 | 97.88 | 96-98 | 0 | Cytochrome b/f complex |
| petB | LSC | 0.037 | 0.004 | 663.06 | 491-710 | 0 | Cytochrome b/f complex |
| psbB | LSC | 0.037 | 0.002 | 1529.04 | 1527-1535 | *Aegilops speltoides* | Photosystem II |
| atpI | LSC | 0.038 | 0.004 | 745.95 | 743-746 | 0 | ATP synthase |
| psaA | LSC | 0.038 | 0.003 | 2255.04 | 2253-2258 | 0 | Photosystem I |
| psbF | LSC | 0.038 | 0.01 | 122.01 | 120-125 | 0 | Photosystem II |
| psbD | LSC | 0.039 | 0.004 | 1063.98 | 1062-1064 | 0 | Photosystem II |
| psbC | LSC | 0.04 | 0.002 | 1424.82 | 1422-1466 | 0 | Photosystem II |
| psbJ | LSC | 0.04 | 0.01 | 125.07 | 123-134 | 0 | Photosystem II |
| rpl23 | LSC | 0.042 | 0.006 | 284.42 | 282-299 | *Aegilops speltoides Secale cereale* | Large ribosomal subunit |
| petD | LSC | 0.044 | 0.005 | 494.83 | 483-566 | 0 | Cytochrome b/f complex |
| psbI | LSC | 0.044 | 0.011 | 117.12 | 111-161 | 0 | Photosystem II |
| ndhJ | LSC | 0.045 | 0.004 | 481.98 | 480-482 | 0 | NADH-dehydrogenase |
| ndhC | LSC | 0.047 | 0.006 | 365.01 | 363-368 | 0 | NADH-dehydrogenase |
| lhbA | LSC | 0.048 | 0.01 | 190.6 | 189-191 | 96 | Photosystem II |
| petA | LSC | 0.048 | 0.004 | 965.07 | 963-974 | 0 | Cytochrome b/f complex |
| ycf4 | LSC | 0.048 | 0.004 | 558.97 | 497-560 | *Festuca arundinacea* | unknown |
| atpA | LSC | 0.049 | 0.003 | 1515.24 | 428-1547 | 0 | ATP synthase |
| ndhE | SSC | 0.049 | 0.006 | 308.01 | 293-338 | 0 | NADH-dehydrogenase |
| rpl14 | LSC | 0.049 | 0.006 | 373.58 | 369-374 | *Hakonechloa macra* | Large ribosomal subunit |
| rps8 | LSC | 0.049 | 0.006 | 411.44 | 395-413 | 0 | Small ribosomal subunit |
| atpB | LSC | 0.05 | 0.003 | 1498.95 | 1488-1502 | 0 | ATP synthase |
| psaC | SSC | 0.05 | 0.009 | 247.98 | 246-248 | 0 | Photosystem I |
| psbN | LSC | 0.05 | 0.013 | 135.54 | 132-296 | 0 | Photosystem II |
| rpoB | LSC | 0.05 | 0.002 | 3229.9 | 3209-3254 | 0 | DNA-dep. RNA polyerase |
| rps19 | IR | 0.05 | 0.007 | 280.86 | 218-305 | *Aegilops cylindrica Anomochloa marantoidea Echinochloa oryzicola Ravenala madagascariensis* | Small ribosomal subunit |
| rps2 | LSC | 0.051 | 0.004 | 713.04 | 701-722 | 0 | Small ribosomal subunit |
| psbA | LSC | 0.052 | 0.004 | 1074.97 | 1062-2207 | *Saccharum NCo 310* | Photosystem II |
| atpF | LSC | 0.053 | 0.005 | 564.64 | 545-569 | 0 | ATP synthase |
| psaI | LSC | 0.053 | 0.013 | 112.92 | 107-113 | 0 | Photosystem I |
| rps14 | LSC | 0.053 | 0.007 | 312.6 | 303-314 | *Festuca arundinacea* | Small ribosomal subunit |
| ndhK | LSC | 0.054 | 0.004 | 746.53 | 671-866 | *Coix lacryma-jobi* | NADH-dehydrogenase |
| rps16 | LSC | 0.054 | 0.007 | 258.38 | 185-281 | *Aegilops speltoides Echinochloa oryzicola* | Small ribosomal subunit |
| rbcL | LSC | 0.055 | 0.004 | 1438.84 | 1409-1466 | 0 | Large subunit rubisco |
| atpE | LSC | 0.057 | 0.006 | 414.9 | 404-419 | 0 | ATP synthase |
| rps4 | LSC | 0.057 | 0.005 | 606.35 | 437-614 | 0 | Small ribosomal subunit |
| ndhI | SSC | 0.058 | 0.005 | 544.92 | 539-551 | 0 | NADH-dehydrogenase |
| ndhH | SSC | 0.059 | 0.004 | 1172.27 | 173-1190 | 0 | NADH-dehydrogenase |
| rps15 | SSC | 0.059 | 0.006 | 265.92 | 200-284 | 0 | Small ribosomal subunit |
| ndhD | SSC | 0.06 | 0.003 | 1505.44 | 1499-1511 | 0 | NADH-dehydrogenase |
| psbK | LSC | 0.06 | 0.01 | 188.9 | 185-242 | 0 | Photosystem II |
| rpl36 | LSC | 0.06 | 0.011 | 115.98 | 114-116 | 0 | Large ribosomal subunit |
| rpoC1 | LSC | 0.061 | 0.003 | 2051.38 | 2027-2075 | 0 | DNA-dep. RNA polyerase |
| psbM | LSC | 0.062 | 0.015 | 107.95 | 105-209 | 0 | Photosystem II |
| rpoA | LSC | 0.064 | 0.004 | 1022.78 | 1016-1061 | 0 | DNA-dep. RNA polyerase |
| ndhG | SSC | 0.065 | 0.006 | 532.52 | 485-533 | 0 | NADH-dehydrogenase |
| rpl16 | LSC | 0.066 | 0.006 | 415.78 | 365-458 | *Hakonechloa macra* | Large ribosomal subunit |
| rps11 | LSC | 0.066 | 0.006 | 432.54 | 417-455 | 0 | Small ribosomal subunit |
| ycf2 | IR | 0.066 | 0.007 | 4801.48 | 572-7184 | 80 | unknown |
| ndhA | SSC | 0.068 | 0.004 | 1084.17 | 917-1097 | 0 | NADH-dehydrogenase |
| psbH | LSC | 0.069 | 0.009 | 224.12 | 222-233 | 0 | Photosystem II |
| rpl20 | LSC | 0.069 | 0.008 | 363.89 | 356-434 | *Secale cereale* | Large ribosomal subunit |
| rpoC2 | LSC | 0.073 | 0.002 | 4447.12 | 2648-4622 | *Brachypodium distachyon* | DNA-dep. RNA polyerase |
| ccsA | SSC | 0.074 | 0.006 | 972.37 | 926-992 | *Arundinaria tecta* | Cytochromee c biogenesis |
| rps12 | IR | 0.074 | 0.007 | 370.95 | 164-404 | 0 | Small ribosomal subunit |
| rps3 | LSC | 0.076 | 0.005 | 702.95 | 657-767 | 0 | Small ribosomal subunit |
| cemA | LSC | 0.077 | 0.005 | 694.75 | 680-701 | 0 | Inner membrane protein |
| rps18 | LSC | 0.079 | 0.008 | 472.56 | 308-515 | *Festuca arundinacea Pharus lappulaceus* | Small ribosomal subunit |
| ycf68 | IR | 0.08 | 0.006 | 398.77 | 221-437 | 56 | unknown |
| rpl33 | LSC | 0.081 | 0.011 | 202.98 | 201-203 | *Pharus lappulaceus* | Large ribosomal subunit |
| clpP | LSC | 0.082 | 0.006 | 649.41 | 614-716 | 0 | ATP-dependent protease |
| ndhF | SSC | 0.087 | 0.004 | 2220.22 | 2153-2240 | *Olyra latifolia* | NADH-dehydrogenase |
| infA | LSC | 0.101 | 0.01 | 308.24 | 234-362 | 0 | Translation initiation factor IF-1 |
| matK | LSC | 0.101 | 0.005 | 1543.63 | 1535-1634 | 0 | Maturase |
| rpl22 | LSC | 0.107 | 0.009 | 441.04 | 308-476 | 0 | Large ribosomal subunit |
| rpl32 | SSC | 0.12 | 0.015 | 184.5 | 161-206 | 0 | Large ribosomal subunit |
| accD | LSC | 0.161 | 0.01 | 1417.43 | 323-1640 | 87 | Acetyl-CoA carboxylase |
| ycf1 | SSC | 0.175 | 0.009 | 4604.41 | 122-5708 | 83 | unknown |
